# Artificial Intelligence Integration With Nanotechnology for Cancer Therapy

**DOI:** 10.1002/mco2.70948

**Published:** 2026-08-30

**Authors:** Shengbin Liu, Zhi Liu, Haixing Shi, Chengyi Wei, Ruijie Zhang, Xiangrong Song

**Affiliations:** ^1^ Department of Critical Care Medicine Frontiers Science Center for Disease‐related Molecular Network State Key Laboratory of Biotherapy and Cancer Center West China Hospital Sichuan University Chengdu China; ^2^ Department of Clinical Medicine Southwest Medical University Luzhou China; ^3^ School of Pharmacy Sichuan University Chengdu China

**Keywords:** AI, cancer therapy, nanomedicine, precision medicine, protein corona

## Abstract

Cancer nanomedicine offers a versatile platform for improving therapeutic index, but its clinical translation remains limited by unpredictable in vivo behavior, heterogeneous biological contexts, and inefficient design paradigms. Artificial intelligence (AI) is emerging as an integrative framework that links data‐driven modeling with nanomedicine design, biological transport, and clinical decision‐making. In this review, we discuss AI‐guided strategies for material design, targeting, payload optimization, and in vivo delivery, with particular attention to protein corona‐mediated biological identity, microenvironment‐responsive activation, and biodistribution modeling. We further examine the translational requirements for AI‐enabled nanomedicine, including data standardization, preclinical learning workflows, clinical stratification and risk‐based governance. Finally, we outline future directions centered on transferable learning architectures, dynamic multiscale modeling and patient‐aware therapeutic strategies. Together, these advances suggest that AI can move cancer nanomedicine beyond empirical formulation toward a more predictive, biologically informed, and clinically responsive discipline.

## Graphical abstract

AI‐enabled nanotechnology provides a continuous learning framework for cancer therapy by integrating nanomaterial design, biological interactions, tumor delivery, clinical data, and therapeutic feedback to support predictive design, patient‐aware optimization, and improved translational decision‐making.

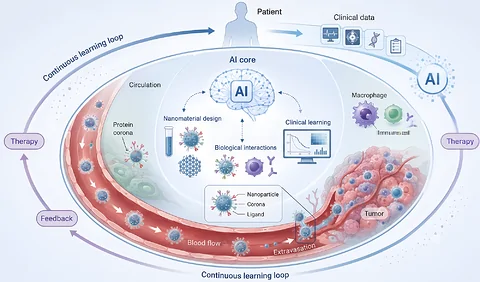

## 1 Introduction

Cancer remains a leading cause of mortality worldwide, and current therapeutic strategies are often constrained by limited specificity, systemic toxicity, and pronounced biological heterogeneity [1, 2, 3, 4]. Nanomedicine offers a promising strategy to address these limitations by enabling controlled drug delivery, improved pharmacokinetics, and targeted therapeutic intervention [5]. However, despite extensive preclinical advances, its clinical impact remains modest and inconsistent [6, 7].

A central challenge lies in the multiscale complexity of nanomedicine systems. Nanomedicine is not simply a formulation problem but a multiscale system involving material properties, interfacial chemistry, targeting behavior, and payload functionality [8, 9]. Importantly, the performance of nanomedicines is not determined solely by their physicochemical properties but also by their dynamic interactions with biological environments, including immune recognition, protein adsorption, vascular transport, and tumor microenvironmental conditions.

Traditional nanomedicine development has largely relied on empirical and iterative optimization, which is inefficient in navigating the high‐dimensional design space defined by these interacting variables [10]. AI offers a fundamentally different approach by enabling data‐driven modeling, prediction, and optimization across multiple levels of the system [11]. Rather than treating design, delivery, and therapeutic response as separate problems, AI provides a framework for integrating these processes into a unified and learnable system.

In this context, AI is best understood as an organizing framework that connects formulation variables, biological interfaces, and therapeutic outcomes. Its value lies in making relationships across these layers more explicit, testable, and reusable, rather than simply adding isolated prediction tools to existing workflows [12].

**FIGURE 1:**
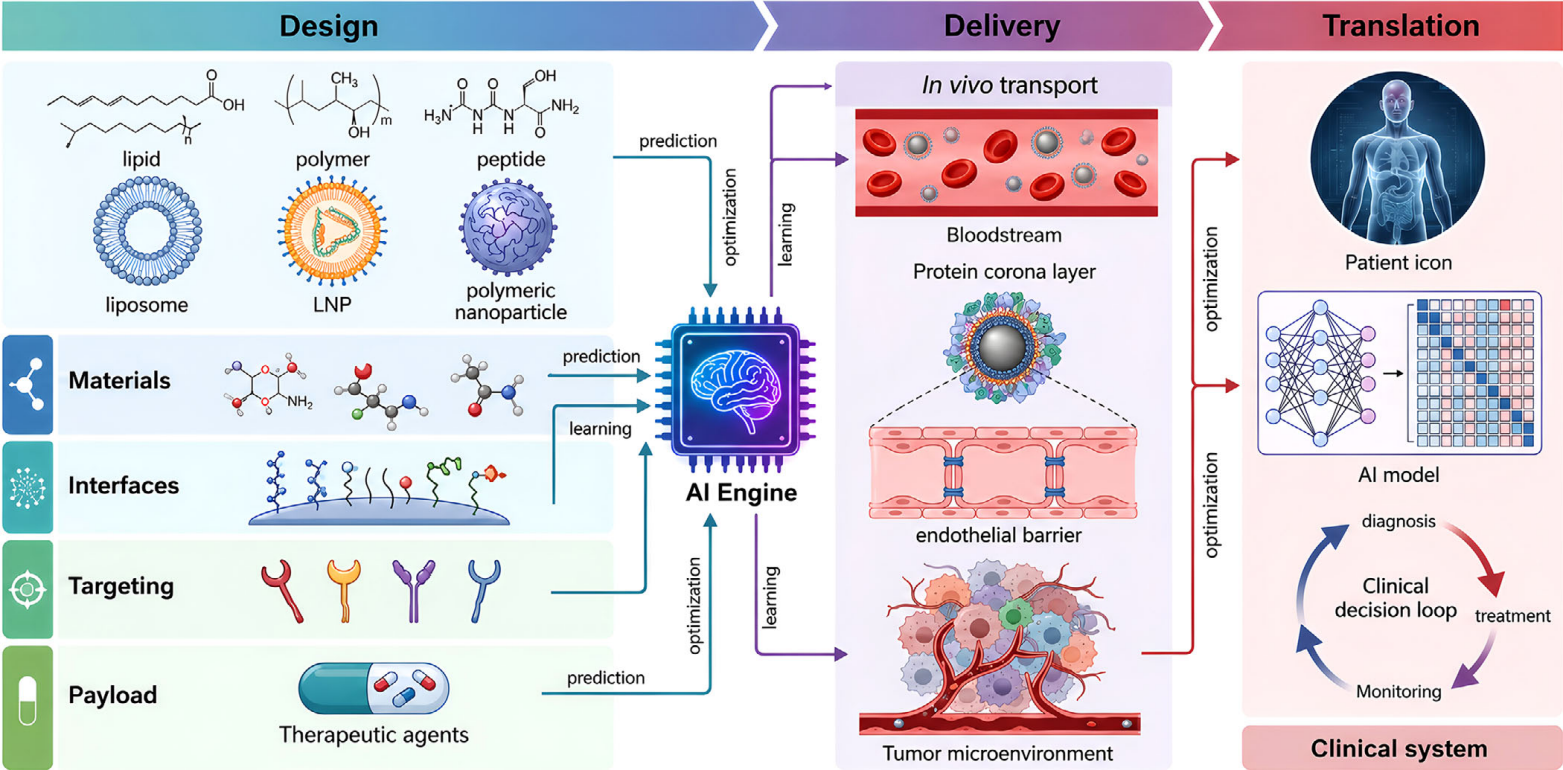
AI‐guided framework for cancer nanomedicine across design, delivery and translation. The schematic summarizes how AI can connect material design, targeting strategies, biological transport, and clinical translation. The figure distinguishes established optimization tasks from emerging model‐guided strategies that still require experimental and clinical validation. Figure created using BioRender.com and Nano Banana 2; final scientific content, labels, and layout were reviewed and edited by the authors.

In this review, we present a framework for AI‐guided nanomedicine in cancer therapy (Figure 1). We first examine how AI reshapes materials, targeting, and payload optimization. We then discuss how AI can decode in vivo transport processes, including protein corona formation, vascular dynamics, and TME interactions. Finally, we address translational requirements, including data quality, learning workflows, clinical stratification and governance, before outlining future directions for precision nanomedicine.

Compared with previous AI‐nanomedicine reviews that primarily catalogue algorithmic applications, this review emphasizes a systems‐level framework that links design variables, biological interfaces, transport behavior, and clinical translation. The added value of this framework is to distinguish established applications from emerging strategies and speculative future directions, while identifying the data, validation, and governance requirements needed for AI‐guided nanomedicine to become clinically credible [13, 14, 15].

## 2 Nanomedicine Design: Materials, Targeting, and Cargo

**FIGURE 2:**
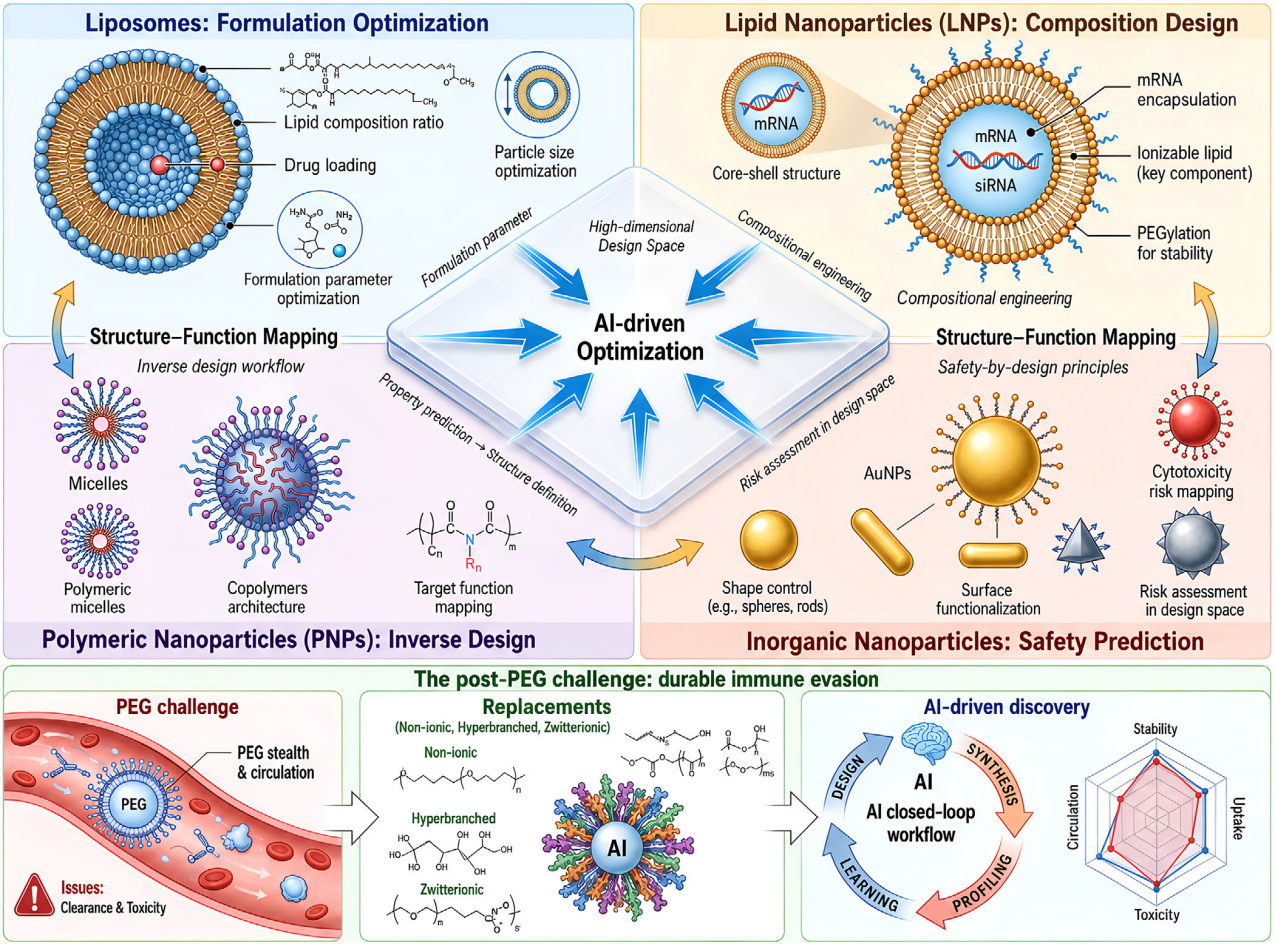
Strategic landscape for AI‐enabled nanomedicine design. The figure summarizes representative material classes and illustrates how AI may support process refinement, formulation optimization, safety‐oriented screening, and post‐PEG stealth design. These applications should be interpreted as model‐guided development strategies rather than clinically established autonomous design systems. Figure created and/or refined using BioRender.com and ChatGPT 5.5 drawing mode; final scientific content, labels, and layout were reviewed and edited by the authors.

The design of cancer nanomedicine involves multiple interconnected components that collectively determine therapeutic performance (Figure 2). These components include materials, targeting strategies, payloads, and biological interactions, all of which are interdependent [16]. As a result, improving individual elements in isolation is often insufficient to achieve meaningful gains in therapeutic performance. AI provides a new approach by enabling integrated and data‐driven optimization across these components [17, 18]. Across different levels of nanomedicine design, AI supports predictive modeling, inverse design, and multiobjective optimization, thereby shifting the field from empirical trial‐and‐error toward systematic‐ and data‐driven engineering [13, 19, 20].

### 2.1 Materials and Formulation Landscapes

Among the principal material classes of nanomedicine, liposomes, lipid nanoparticles (LNPs), polymeric nanoparticles, and inorganic nanoparticles represent distinct but complementary arenas for AI‐enabled design and formulation. Representative examples of AI‐enabled design and formulation across these platforms are summarized in Table 1. Liposomes remain the most mature platform, with the longest clinical track record and the broadest oncological use [21, 22, 23]; accordingly, AI is being applied less to reinvent the platform than to refine it, particularly through microfluidic process optimization, manufacturing control, and prediction of critical quality attributes such as particle size and formulation robustness [24]. By contrast, LNPs have become the most active testbed for AI‐driven materials innovation, largely because their performance depends on navigating a high‐dimensional design space defined by ionizable lipid chemistry, helper‐lipid composition, and multicomponent interactions [25, 26, 27]. In this setting, machine learning (ML) is increasingly used to prioritize lipid candidates, optimize compositional balance and improve encapsulation, stability and intracellular delivery [28, 29, 30, 31]. Polymeric nanoparticles occupy a different position: their appeal lies in the extraordinary tunability of polymer chemistry, architecture and release behavior, but this same flexibility creates a highly multidimensional formulation problem [32, 33, 34]. Here, AI is particularly valuable for linking physicochemical descriptors and process variables to formulation outcomes, thereby enabling inverse optimization of particle size, drug loading, surface properties and release kinetics while reducing empirical trial‐and‐error. Inorganic nanoparticles, in turn, offer unique optical, magnetic, and structural functionalities that make them especially attractive for imaging, radiosensitization, and theranostic applications, yet their broader translation remains constrained by biosafety concerns, including persistence, limited biodegradability, and off‐target toxicity [35]. In this context, AI has begun to shift from performance optimization alone toward structure‐toxicity and biodistribution modeling, helping define safer design windows across core composition, size, shape, and surface chemistry [36]. Taken together, these four classes illustrate that the role of AI in nanomedicine is not uniform across materials: in clinically mature systems it increasingly improves manufacturability and reproducibility, whereas in more chemically or structurally open‐ended platforms it functions as a tool for compressing design space, prioritizing candidates and balancing functional performance with translational feasibility.

**TABLE 1:** Representative studies supporting AI‐enabled design and formulation across major nanomedicine material classes.

Platform	Model type	Task/outcome	Dataset size or source	Validation approach	Experimental level	Major limitation	Translational readiness
LNP / ionizable lipid	Geometry‐informed ML	Organ‐selective mRNA delivery; spleen‐targeting candidates [49]	Experimentally generated lipid/formulation library	In vivo organ delivery validation	Preclinical in vivo	Dataset and chemistry‐space dependence	Emerging preclinical
LNP/ionizable lipid	Review/predictive modeling	AI‐guided lipid discovery and formulation optimization [50]	Published LNP datasets	Literature‐level synthesis	Preclinical evidence base	Not a single validated model	Conceptual/emerging
LNP/ionizable lipid	ML‐guided virtual screening	Lead lipid discovery for mRNA delivery [51]	Combinatorial lipid library	Benchmark comparison and in vivo validation	Preclinical in vivo	Limited external validation	Emerging preclinical
LNP/ionizable lipid	In silico screening + ML	Pulmonary gene‐delivery LNP design [52]	Large virtual library plus experimental screen	In vitro and in vivo validation	Preclinical in vivo	Translation limited by safety and manufacturability	Emerging preclinical
LNP/ionizable lipid	Virtual screening/regression	pKa and delivery‐efficiency prediction [30]	Virtual chemical space and experimental subset	Experimental validation of selected candidates	Preclinical	Model transferability uncertain	Discovery‐stage
Liposome	DoE‐assisted ML	Microfluidic process optimization [24]	Designed experimental runs	Cross‐validation / confirmatory batches	In vitro formulation	Scale‐up validation limited	Process‐development ready
Liposome	ML formulation prediction	Composition‐process‐performance mapping [53]	Published and experimental formulation data	Model testing on held‐out formulations	In vitro formulation	Endpoint‐specific models	Preclinical formulation
LNP	ML‐assisted formulation optimization	mRNA/siRNA co‐delivery optimization [31]	Formulation screen	In vitro/in vivo functional validation	Preclinical in vivo	Indication‐specific validation	Emerging preclinical
LNP	Transformer‐based neural network	Composition design across formulation space [54]	Large formulation dataset	Experimental validation of predicted LNPs	Preclinical	Requires comparable training distributions	Emerging preclinical
LNP	AI‐assisted screening	Brain‐targeted mRNA LNP design [55]	Screening and validation dataset	In vivo targeting validation	Preclinical in vivo	Clinical brain delivery not established	Emerging preclinical
LNP	Deep learning	mRNA‐delivery optimization [56]	LNP development dataset	Experimental validation of candidates	Preclinical	Dataset sparsity and domain shift	Emerging preclinical
PLGA nanoparticle	DoE + ML	Nanoprecipitation optimization [57]	DoE formulation matrix	Confirmatory experiments	In vitro formulation	Limited biological validation	Formulation‐development ready
Nanocomposite	Physics‐informed ML	Gemcitabine‐loaded nanocomposite design [58]	Simulation/experimental descriptors	Predictive testing	In vitro/computational	Mechanistic assumptions require validation	Discovery‐stage
Polymeric micelle	ML regression	Drug‐loading prediction [59]	Polymer‐drug loading dataset	Cross‐validation	Computational / in vitro	Limited in vivo relevance	Early formulation screening
Polymeric Long‐acting injectable	ML modeling	Depot formulation selection [60]	Polymeric injectable dataset	Held‐out validation	Formulation/preclinical	Cancer‐specific translation uncertain	Formulation‐development ready
Inorganic nanoparticle	Large‐scale ML	Structure‐outcome mapping in cancer nanomedicine [36]	Large preclinical literature‐derived dataset	Model evaluation across curated studies	Preclinical meta‐analysis	Literature bias and heterogeneous endpoints	Design‐rule generation
Inorganic nanoparticle	ML toxicity prediction	Cellular toxicity screening [61]	Nanotoxicity dataset	Cross‐validation/test‐set evaluation	In vitro toxicity	Limited organism‐level prediction	Safety‐screening support
Inorganic nanoparticle	Mechanistic modeling + ML	Safer inorganic nanoparticle design [62]	Mechanistic descriptors and experimental data	Model‐informed experimental validation	Preclinical	Requires broader external validation	Safety‐oriented preclinical

Abbreviations: DoE, design of experiments; PLGA, poly‐(lactic‐co‐glycolic acid).

Polyethylene glycol (PEG) has long been the canonical stealth coating in nanomedicine because its hydrated surface suppresses nonspecific protein adsorption, reduces opsonization, and prolongs circulation. However, repeated exposure to PEGylated materials can induce anti‐PEG antibodies, accelerate blood clearance, and amplify complement activation, making it increasingly clear that the post‐PEG challenge is not merely to replace one polymer with another, but to engineer surface chemistries that achieve durable immune evasion in vivo [37]. Several candidate material classes are beginning to define this transition, including nonionic PEG analogues such as poly‐(oligo‐(ethylene glycol) methyl ether methacrylate), hyperbranched polymers such as polyglycerol, and zwitterionic polymers that form exceptionally stable hydration shells [38, 39, 40, 41]. Yet recent evidence also indicates that stealth cannot be inferred from antifouling chemistry alone: even unPEGylated liposomes may acquire prolonged circulation when they recruit coronas depleted in opsonic components. The central design question therefore shifts from which coating appears most inert in isolation to which surface chemistry most consistently generates a protective immune interface in vivo. In this context, AI is particularly valuable because post‐PEG stealth design is a high‐dimensional inverse problem involving nonlinear and often competing objectives, including hydration, protein adsorption, complement activation, phagocytic uptake, circulation half‐life, and repeat‐dose tolerability [42, 43, 44]. Generative and inverse‐design models can expand the accessible space of immune‐evasive polymers, but their greatest value will likely emerge when coupled to closed‐loop design‐synthesis‐test‐learn workflows [45], in which candidate materials are iteratively generated, synthesized, profiled, and fed back into active‐learning models [46, 47, 48]. Under this framework, stealth‐material discovery may shift from empirical PEG substitution towards predictive discovery of clinically relevant immune‐evasive surface chemistries.

### 2.2 Engineered Targeting Modalities

**FIGURE 3:**
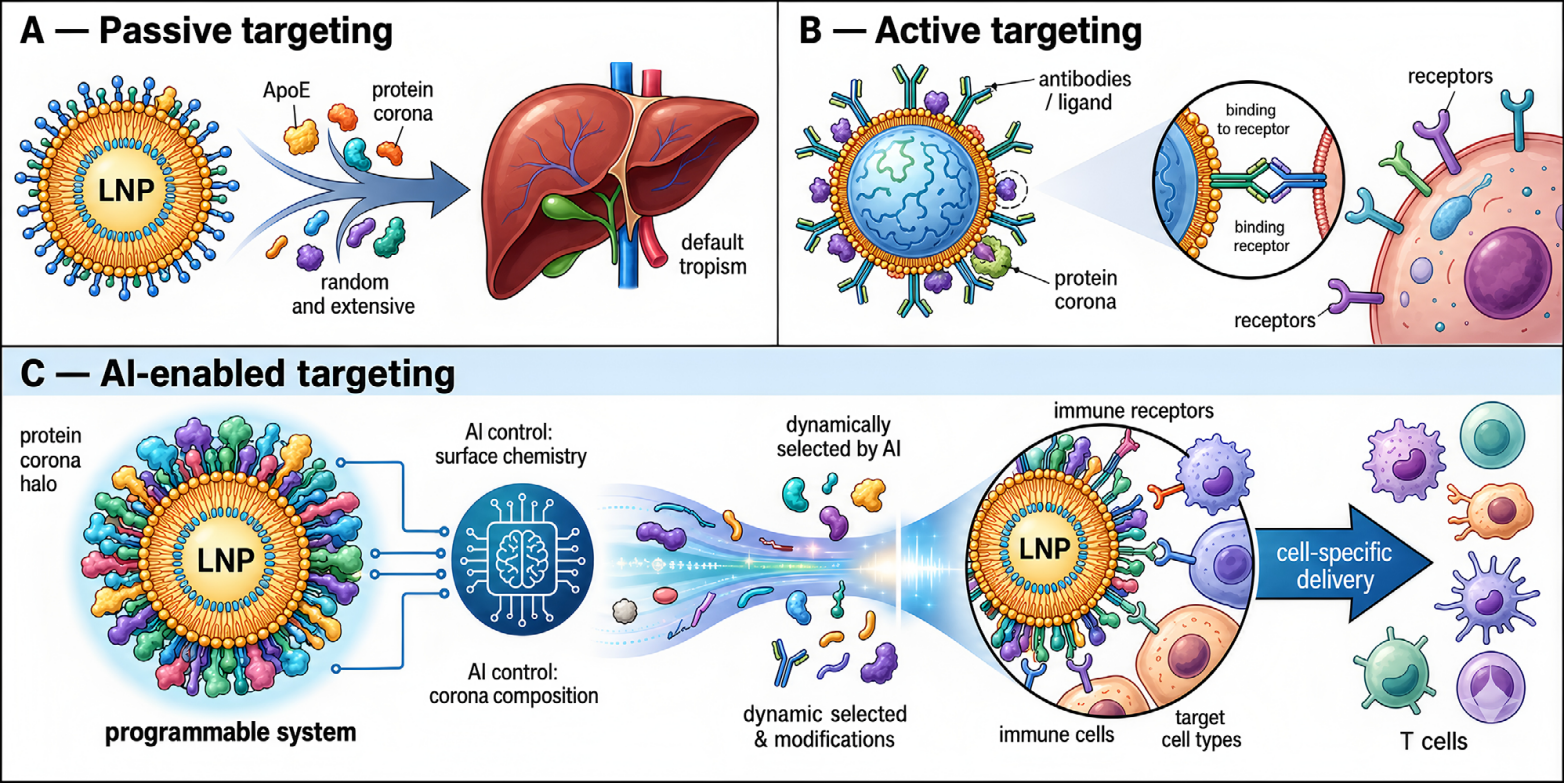
Conventional LNP targeting strategies and emerging AI‐enabled targeting. (A) Passive targeting mediated by endogenous protein adsorption and default organ tropism. (B) Ligand‐mediated active targeting. (C) An emerging model‐guided concept in which surface chemistry and corona composition may be tuned to improve cell‐selective delivery; this remains primarily preclinical. Figure created and/or refined using BioRender.com and ChatGPT 5.5 drawing mode; final scientific content, labels, and layout were reviewed and edited by the authors.

Targeting in nanomedicine should not be viewed as a binary distinction between passive and active strategies, but as a continuum of programmable delivery logic shaped by material properties, biological interactions, and engineered recognition. In this framework, nanoparticle biodistribution and cellular uptake emerge from the interplay between intrinsic physicochemical features and extrinsic biological environments, both of which can be systematically modulated (Figure 3).

Passive targeting represents an intrinsic delivery logic arising from nanoparticle‐host interactions rather than a purely incidental phenomenon [63]. A canonical example is the hepatotropism of lipid nanoparticles (LNPs), which is largely mediated by adsorption of endogenous apolipoproteins and subsequent receptor‐dependent uptake (Figure 3A). Importantly, such tropism is not fixed but can be reprogrammed through rational modification of nanoparticle composition, particularly ionizable lipid structure and multicomponent interactions [64]. This observation suggests that so‐called passive targeting is, at least in part, a designable and controllable property.

AI enables a shift from empirical observation of biodistribution toward predictive and programmable tropism engineering. By learning structure‐distribution relationships from large datasets, AI models can identify key molecular and compositional determinants governing organ selectivity [65]. This capability opens the possibility of designing nanocarriers with tailored delivery profiles that extend beyond default accumulation patterns, thereby expanding the accessible targeting landscape (Figure 3C).

Active targeting strategies introduce additional layers of specificity through engineered ligands, including antibodies, peptides, and aptamers (Figure 3B). Among these, antibody‐guided targeting has emerged as a particularly powerful approach for cell‐selective delivery, especially in the context of immunotherapy and in vivo cell engineering [66]. For example, targeted LNP systems have been developed to selectively deliver genetic payloads to T‐cell subsets, enabling in vivo CAR‐T generation [67, 68, 69]. However, conventional ligand design often focuses on affinity and binding specificity without fully accounting for downstream functional outcomes such as internalization efficiency, intracellular trafficking, and cell‐state selectivity (Figure 3B).

AI provides an opportunity to redefine active targeting beyond affinity optimization (Figure 3C). By integrating structural, functional, and cellular data, AI‐driven approaches can enable the design of ligands against functionally relevant epitopes and specific cellular states [70]. Recent advances in de novo antibody design and affinity maturation further demonstrate the potential of AI to expand the accessible design space and optimize targeting performance at multiple levels [71, 72, 73].

Aptamers represent a complementary targeting modality with advantages in chemical synthesis, tunability, and potential tissue penetration [74, 75]. However, their clinical translation remains limited by challenges in stability, specificity, and in vivo performance. To date, only two aptamer therapeutics, pegaptanib and avacincaptad pegol, have been approved, and neither is an anticancer drug [76]. In oncology, exemplars such as AS1411 and NOX‐A12 have helped define the field but also illustrate its challenges. AS1411 continues to be hindered by uncertainty surrounding its precise mechanism of action, whereas NOX‐A12 has a clearer mechanistic basis as a CXCL12‐targeting Spiegelmer but still faces translational questions related to efficacy boundaries, indication selection, and combination strategies [77, 78, 79].

AI‐driven sequence‐structure‐function modeling may substantially improve aptamer discovery efficiency by reducing experimental screening burden and enabling rational optimization of binding kinetics, structural stability, and resistance to biological degradation. SELEX is inherently a high‐dimensional iterative enrichment process and is therefore well suited to machine‐learning intervention [80]. Recent work has shown that AI can help build sequence‐structure‐function models capable of predicting affinity, specificity, and conformational stability before experimental validation, thereby reducing the number of screening rounds and lowering empirical cost [81, 82]. Coupled with generative models and active learning, this creates the prospect of a closed‐loop framework linking computational prediction, experimental validation, and model updating. Because aptamers are nucleic‐acid‐based molecules, they are also amenable to AI‐driven sequence optimization strategies analogous to those used for RNA therapeutics; however, the objective functions are different [83]. Rather than prioritizing translation efficiency or avoidance of innate immune sensing, aptamer optimization should focus more strongly on binding constants, kon/koff kinetics, specificity, resistance to interference, preservation of functional conformation under tumor‐microenvironment conditions, and improved resistance to nuclease degradation and renal clearance [84].

Collectively, these developments suggest that targeting in nanomedicine is evolving from a ligand‐centric paradigm toward an AI‐guided, system‐level design problem. In this emerging framework, targeting is not defined solely by surface ligands but by the coordinated interaction between material properties, biological context, and functional delivery outcomes.

### 2.3 Payload Optimization

The therapeutic payload is a central determinant of nanomedicine efficacy, defining not only the mechanism of action but also influencing delivery requirements, intracellular trafficking, and overall therapeutic outcome. Conventional development strategies often treat payload and carrier as separable components, optimizing each independently. However, this sequential approach overlooks the strong coupling between payload properties and carrier design, which jointly determine pharmacokinetics, biodistribution, and biological activity. AI provides a framework for integrating these interdependencies by enabling co‐optimization of payload and carrier within a unified design space. Rather than optimizing delivery vehicles in isolation, AI‐driven approaches allow simultaneous consideration of payload stability, release kinetics, targeting requirements, and biological response, thereby improving overall system performance [85].

For small‐molecule therapeutics, AI is reshaping both molecular generation and molecular optimization. Recent advances indicate that generative models can support not only the creation of novel chemical entities, but also a broader spectrum of practical molecular design tasks that more closely reflect real medicinal‐chemistry workflows [86]. At the same time, AI frameworks for candidate prioritization, including those coupled to molecular docking, quantum‐chemical properties and other high‐fidelity scoring or oracle functions, are becoming increasingly important because they can reduce the time and computational cost associated with traditional high‐accuracy simulations [87]. More broadly, the field is moving toward unified or foundation‐model‐like molecular frameworks capable of supporting multiple downstream tasks within a single modeling system, including representation learning, property prediction and molecular generation [88]. Targeted protein degradation represents another payload modality of growing relevance to cancer therapy. By delivering PROTACs or related degrader systems, it may become possible to intervene in oncogenic and immune‐evasion‐associated pathways that are difficult to modulate through conventional inhibition alone [89]. Here again, AI is well positioned to accelerate design by improving ligand selection, docking‐informed ternary‐complex modeling, linker optimization, and multiparametric developability assessment [90].

AI is also beginning to expand the design space for peptide and protein therapeutics [91]. Rather than relying exclusively on low‐throughput, stepwise screening strategies, machine‐learning‐guided workflows increasingly enable direct exploration of sequence‐structure space, thereby improving hit discovery and broadening the range of accessible candidates [92]. In oncology and cancer immunotherapy, this raises the prospect of designing peptides and proteins with improved structural quality, target engagement and functional performance. Although this direction remains dominated by preclinical progress, recent work suggests that AI‐driven design may substantially accelerate the development of functional peptide therapeutics, including candidates directed against immuno‐oncology targets.

Among biologic payloads, monoclonal antibodies have become one of the most important therapeutic classes in oncology, and AI is now pushing antibody discovery beyond conventional library screening toward structure‐constrained generative design [72]. Recent studies have shown that AI can support de novo antibody design against specified epitopes, while parallel computational workflows are enabling high‐throughput generation and prioritization of epitope‐specific antibody candidates [93]. When combined with advances in antibody structure prediction, these methods substantially enlarge the explorable design space for therapeutic antibodies and may ultimately support more precise optimization of affinity, specificity, and developability [94]. Emerging work further suggests that AI may help address downstream formulation‐relevant liabilities, such as viscosity, thereby linking molecular design to manufacturability and delivery feasibility [95].

For nucleic acid therapeutics, one of the most mature AI applications remains sequence optimization. In the case of mRNA, major constraints still include insufficient translation efficiency, limited expression durability, and the need to balance potency with stability and immune compatibility. Recent studies have used large‐scale ribosome profiling and related datasets to learn the relationship between sequence and translation output, and have combined these models with generative optimization to identify improved codon usage patterns across much broader sequence spaces [96, 97]. Beyond coding‐region optimization, AI is increasingly being extended to untranslated regulatory regions, including the 5′ and 3′ UTRs, and to predictive frameworks for translation efficiency more generally [98, 99]. Similar strategies are also being applied to siRNA design and silencing‐efficiency prediction [100, 101]. Importantly, the optimization objectives for nucleic acid payloads are inherently multidimensional, spanning expression, stability, immunogenicity, intracellular processing and in vivo fate; this makes them especially well‐suited to multiobjective modeling rather than single‐parameter optimization.

AI may also act as a catalyst for the optimization of CAR payloads. For in vivo CAR‐T approaches in particular, efficacy and safety depend not only on delivery selectivity but also on the quality of the encoded genetic cargo. Models trained on large protein sequence and structure datasets may eventually support the joint optimization of antigen‐recognition domains, hinge and transmembrane regions, and downstream signaling modules, in the future, to improve potency and specificity while minimizing off‐target activation, tonic signaling, and functional exhaustion [102].

Taken together, these developments highlight that payload design cannot be decoupled from carrier engineering. Instead, therapeutic function emerges from the coordinated interaction between payload properties, nanocarrier structure, and biological context. In this framework, AI enables a shift from sequential optimization toward integrated co‐design, allowing nanomedicine to be engineered as a unified therapeutic system rather than a combination of independent components.

### 2.4 Micro/Nanorobotic Therapeutics: An Emerging Preclinical Frontier

Micro‐ and nanorobotic therapeutics represent an emerging branch of nanomedicine in which nanoscale or microscale constructs are engineered to perform guided movement, stimulus‐responsive actuation, molecular recognition, or spatially localized therapeutic functions. Compared with conventional nanocarriers, which mainly rely on passive transport, vascular permeability, ligand–receptor recognition, or endogenous biological interactions, micro‐/nanorobotic systems attempt to introduce an additional layer of physical or molecular control. However, the term “nanorobot” should be used cautiously. Most current platforms are not fully autonomous medical robots, but DNA‐based molecular devices, magnetically actuated micro‐/nanoswimmers, bacteria‐driven biohybrid microrobots, or stimuli‐responsive constructs that operate under constrained experimental conditions [103, 104, 105, 106, 107].

Several preclinical studies support the feasibility of this concept, although they do not yet establish clinical maturity. Early DNA nanorobotic systems demonstrated logic‐gated transport of molecular payloads and provided a conceptual basis for stimulus‐responsive nanoscale devices [103]. In cancer models, a nucleolin‐responsive thrombin‐loaded DNA nanorobot was shown to open in response to tumor‐associated endothelial cues, expose thrombin locally, induce intratumoral thrombosis, and inhibit tumor growth in mice, with additional safety evaluation in mice and Bama miniature pigs [104]. More recently, a pH‐responsive DNA robotic switch was designed to hide cytotoxic ligand patterns at physiological pH and expose them under acidic tumor‐like conditions, thereby promoting death‐receptor clustering and reducing breast‐cancer xenograft growth in mice [105]. These studies indicate that molecularly programmed DNA nanodevices can execute conditional therapeutic actions in vivo, but they should be interpreted as preclinical proof‐of‐concept platforms rather than clinically validated autonomous nanorobots.

Biohybrid micro‐/nanorobots provide another line of evidence. Bacteria‐based microrobots have been explored as motile carriers that exploit bacterial chemotaxis toward tumor‐derived gradients, with tumor‐localized fluorescence signals observed in mouse tumor models [106]. Magneto‐aerotactic bacteria have also been used to transport drug‐loaded nanoliposomes into hypoxic regions of colorectal tumor xenografts under magnetic guidance, illustrating how biological motility and external‐field control can be combined to improve spatial delivery in selected experimental settings [108]. These studies are important because they address a major limitation of passive nanocarriers—poor penetration into hypoxic and poorly vascularized tumor compartments—but their experimental contexts remain limited, and their translation will require stringent control of bacterial safety, immunogenicity, biodistribution, clearance, and manufacturing reproducibility.

AI is relevant to this field primarily as an enabling design, navigation, and control tool rather than a clinically autonomous therapeutic controller. Machine‐learning models can assist in optimizing microrobot geometry, material composition, propulsion parameters, magnetic actuation, cargo loading, and release behavior [109]. Deep‐learning‐based planning has also been used to guide microrobot swarm navigation and cargo transport in structured experimental environments, supporting the feasibility of computationally assisted distribution planning and adaptive navigation [110]. In future cancer applications, similar approaches may help integrate imaging feedback, trajectory planning, and actuation control; however, these capabilities remain largely engineering‐stage or preclinical and have not yet been validated as autonomous cancer therapies in patients.

Major barriers continue to limit near‐term translation. Micro‐/nanorobots must function in blood, mucus, interstitial matrix, and heterogeneous tumor tissues, where flow, viscosity, Brownian motion, immune clearance, protein adsorption, extracellular matrix confinement, and heterogeneous oxygen or pH gradients can disrupt propulsion and targeting. Additional unresolved issues include biocompatible power sources, potential fuel toxicity, payload capacity, controllable release, degradation and clearance, off‐target accumulation, real‐time imaging depth, swarm controllability, sterilization, scalability, batch‐to‐batch reproducibility, and regulatory classification [111, 112, 113]. Therefore, micro‐/nanorobotic therapeutics should currently be viewed as an exploratory preclinical direction within AI‐enabled nanomedicine. Their future translational value will depend on whether AI‐guided design, quantitative imaging, closed‐loop actuation, and rigorous safety evaluation can converge into reproducible systems with clear advantages over optimized nanocarriers.

## 3 Decoding In Vivo Transport: From Biological Identity to Delivery Logic

In vivo delivery can be understood across three interconnected layers: biological identity, transport barriers, and microenvironmental logic. Immunotherapy has transformed the treatment landscape of cancer by harnessing the host immune system to control disease, delivering durable clinical benefit in settings in which conventional modalities often fail [114]. Although systemically administered immunotherapies, including immune checkpoint blockade, bispecific antibodies, and adoptive T cell therapies, have achieved notable clinical success, with particularly striking efficacy in several hematological malignancies, durable benefit remains elusive for most solid tumors. Moreover, in hard‐to‐treat and metastatic disease, the systemic delivery of immunomodulatory agents is frequently associated with off‐target inflammation and dose‐limiting toxicities, thereby constraining therapeutic efficacy and raising substantial safety concerns [115]. Achieving precise tumor targeting while limiting systemic toxicity has therefore emerged as a central challenge in cancer immunotherapy.

More broadly, directing nanocarriers and nanomedicines to their intended sites of action remains an enduring challenge in modern medicine. In cancer, however, this problem is amplified by the abnormal TME, metastatic dissemination, and pronounced inter‐ and intratumor heterogeneity. Deciphering the in vivo fate of nanomedicines in tumors, from systemic circulation and biodistribution to tumor accumulation, cellular uptake, and clearance, will therefore be essential for accelerating the development of precision immunotherapeutic strategies with improved targeting, enhanced efficacy, and reduced toxicity.

### 3.1 Protein Coronas and Endogenous Targeting

**FIGURE 4:**
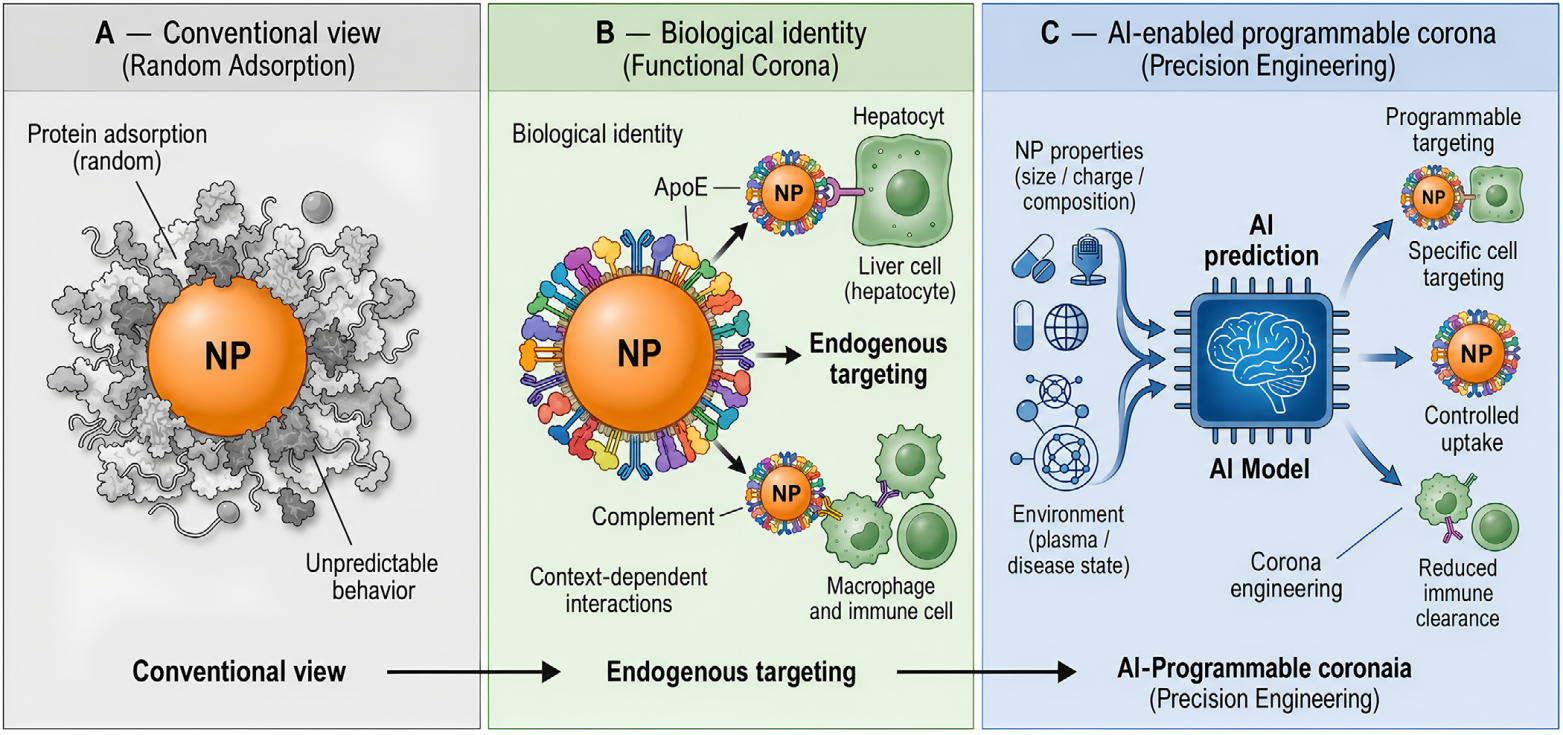
AI‐enabled analysis of protein corona for endogenous targeting in nanomedicine. (A) The conventional view of protein adsorption as a source of unpredictable biological identity. (B) The corona as a context‐dependent biological interface. (C) Illustration of how AI may help predict corona composition and nominate surface designs that recruit favorable protein patterns. The term programmable indicates a design objective rather than a clinically established capability. Figure created using BioRender.com and Nano Banana 2; final scientific content, labels, and layout were reviewed and edited by the authors.

The protein corona should be understood not merely as a byproduct of nanoparticle exposure to biological fluids, but as a dynamic and information‐rich interface that encodes biological identity and governs in vivo fate [116, 117]. Upon entering biological environments, nanoparticles rapidly adsorb proteins and other biomolecules, forming a corona layer that redefines their interactions with cells, tissues, and the immune system. As a result, nanoparticle behavior in vivo is often dictated less by its original design than by the biological identity acquired through corona formation (Figure 4A).

Unlike engineered targeting ligands, which impose exogenous recognition motifs, the protein corona represents an endogenous targeting system that emerges from host biology [118]. This system is inherently context‐dependent and can redirect nanoparticles toward specific cell types or tissues based on the composition of adsorbed biomolecules [119]. A well‐established example is the role of apolipoprotein E (ApoE) in mediating hepatic uptake of LNPs through receptor‐dependent pathways [120]. Such mechanisms illustrate how endogenous biological processes can be harnessed to achieve efficient and selective delivery. Beyond hepatotropic delivery, emerging evidence suggests that protein coronas may also contribute to preferential interactions with immune cell populations, including macrophages and other myeloid cells (Figure 4B). These interactions are likely governed by complex combinations of complement proteins, opsonins, and immune‐associated factors, rather than single molecular determinants [121, 122, 123]. However, the underlying principles remain poorly understood due to the high dimensionality and context dependence of corona formation [124].

AI provides a unique opportunity to transform protein corona research from descriptive proteomics into predictive and ultimately programmable biology. ML models can quantitatively predict corona composition from nanoparticle physicochemical properties and environmental variables, outperforming conventional linear approaches [125, 126, 127, 128]. More importantly, these models can link predicted corona profiles to functional outcomes, including cellular uptake, immune activation, and biodistribution patterns (Figure 4C).

This predictive capability enables a conceptual shift from avoiding protein adsorption to engineering it. Rather than minimizing corona formation, nanoparticles can be designed to selectively recruit functionally advantageous biomolecular layers that promote desired biological interactions [129, 130]. In this framework, the protein corona becomes a tunable interface through which nanomedicine can engage endogenous transport pathways and immune systems [131]. Ultimately, the protein corona may represent a programmable biological interface that bridges material design and in vivo function [132]. By integrating AI‐driven prediction with experimental validation, it may become possible to design nanocarriers that harness host biology for targeted and context‐specific delivery, thereby transforming an historically unpredictable phenomenon into a controllable component of nanomedicine.

### 3.2 Vascular Transport and Biological Barriers

**FIGURE 5:**
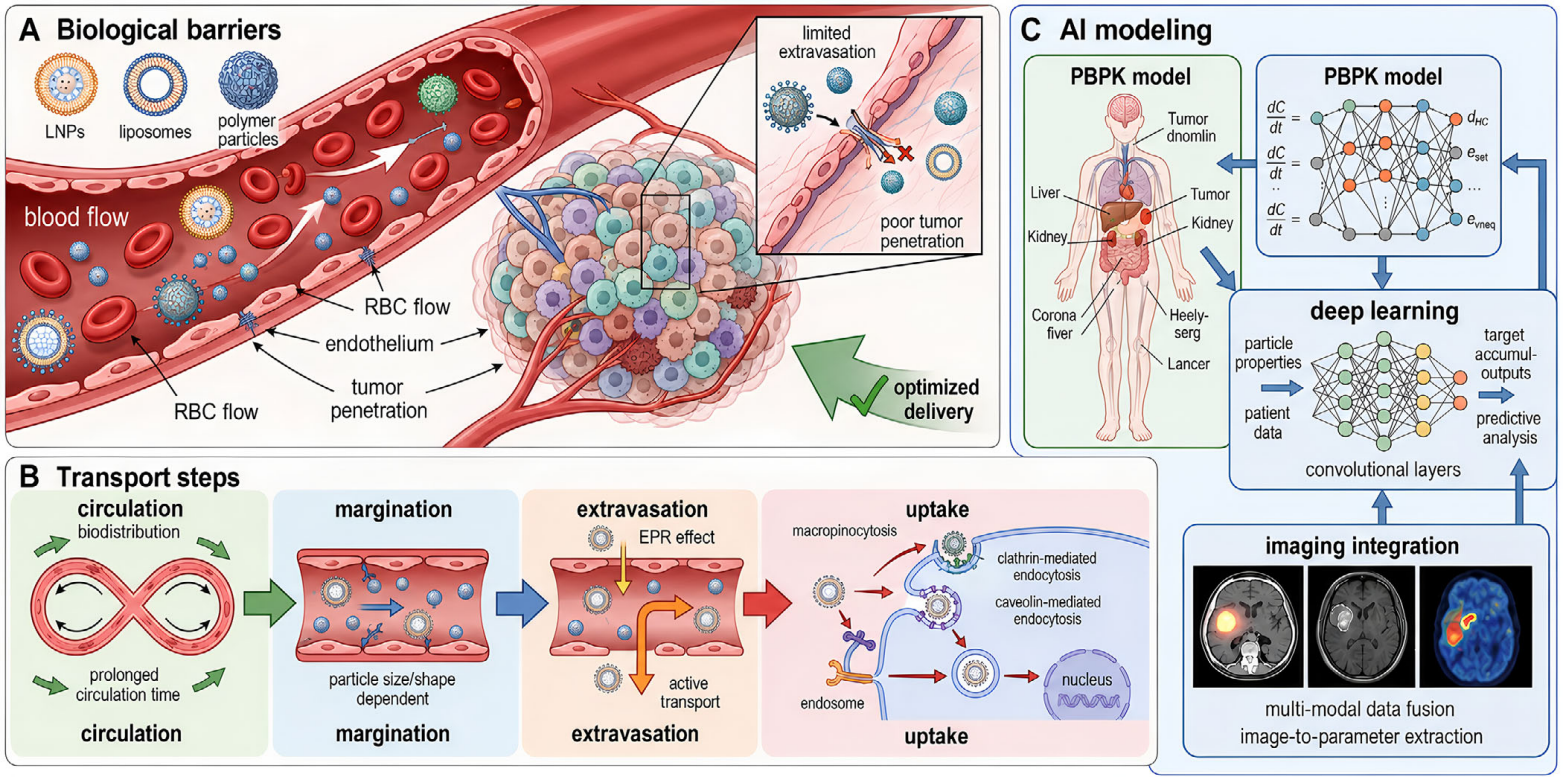
Nanoparticle in vivo transport, biological barriers and AI‐assisted optimization. (A) Biological barriers that limit nanoparticle distribution. (B) Transport steps including circulation, margination, extravasation and cellular uptake. (C) Illustration of how computational models may integrate physicochemical descriptors, physiological data, and imaging readouts to support hypothesis generation and delivery prediction. Figure created and/or refined using BioRender.com and ChatGPT 5.5 drawing mode; final scientific content, labels, and layout were reviewed and edited by the authors.

Hemodynamics is an upstream, yet often underappreciated, determinant of nanomedicine delivery and may represent one of the primary physical constraints on vascular transport (Figure 5A,B). Nanoparticles do not approach the vessel wall freely. Instead, they are first subjected to flow‐induced spatial segregation, in which the red‐blood‐cell‐dominated core flow redistributes suspended particles and limits their lateral migration towards the endothelium [133]. In tumors, this problem is further compounded by pathological vascular remodeling, irregular perfusion and heterogeneous shear environments, which together produce highly nonuniform patterns of nanoparticle margination, wall interaction and local deposition. These considerations suggest that nanomedicine design must extend beyond composition and receptor targeting to incorporate particle geometry, deformability, adhesive behavior and shear responsiveness as central determinants of vascular transport [134, 135, 136].

At its core, this is a coupled physical–biological problem. The opportunity for AI lies not in replacing known transport physics, but in integrating it with experimental data and biologically complex settings. By embedding constraints related to fluid dynamics, mass transfer, particle mechanics, wall interactions, and transendothelial flux into data‐driven frameworks, AI‐enabled models could support more predictive descriptions of margination, endothelial adhesion, extravasation, and local deposition [137]. In turn, such hybrid modeling strategies may enable inverse nanoparticle design tailored to specific flow fields, vascular architectures, and lesion ecologies.

The blood–brain barrier (BBB) provides one of the most stringent test cases for this paradigm. As a highly regulated neurovascular unit, the BBB maintains central nervous system homeostasis, supports normal neural function, and protects the brain from toxins and pathogens. These same properties, however, render the treatment of central nervous system malignancies, particularly brain tumors such as glioma, exceptionally challenging [138]. Physiologically relevant BBB models are therefore essential for understanding the selective transport properties of the barrier and for improving the translational relevance of future drug‐development efforts. Recent advances in BBB organoids, neurovascular‐unit models, and other three‐dimensional in vitro systems are beginning to provide such platforms [139].

Here again, the combination of AI and organoid technologies is particularly compelling. The assembly of BBB organoids is inherently a high‐dimensional combinatorial problem involving cell ratios, extracellular‐matrix composition, morphogen cues, developmental timing, and hemodynamic variables [140]. Their evaluation is similarly multimodal, spanning single‐cell transcriptomics, proteomic readouts, tight‐junction architecture, transendothelial electrical resistance, permeability measurements, and spatial organization [141]. Accordingly, the principal value of AI is not merely to accelerate optimization, but to infer structure–function relationships within the neurovascular unit and predict how spatial organization governs barrier performance (Figure 5C). When coupled with temporal modeling and digital‐twin frameworks, AI may further enable simulation of BBB development, maturation, and homeostatic maintenance, thereby supporting dynamic control over organoid trajectories rather than endpoint‐only characterization [142].

This advantage is not unique to the BBB. Across organoid systems more broadly, the deeper contribution of AI may lie less in efficiency gains than in its ability to uncover the generative rules of complex biological organization (Figure 5C). As recent analyses have emphasized, the convergence of AI, organoid platforms, and high‐content phenotyping is beginning to shift brain delivery and other targeted‐delivery research from empirical exploration toward cross‐scale, predictive design with improved in vivo relevance [143]. In that sense, AI may help transform advanced in vitro models from screening tools into mechanistic engines for translational nanomedicine.

### 3.3 The TME as Delivery Logic

**FIGURE 6:**
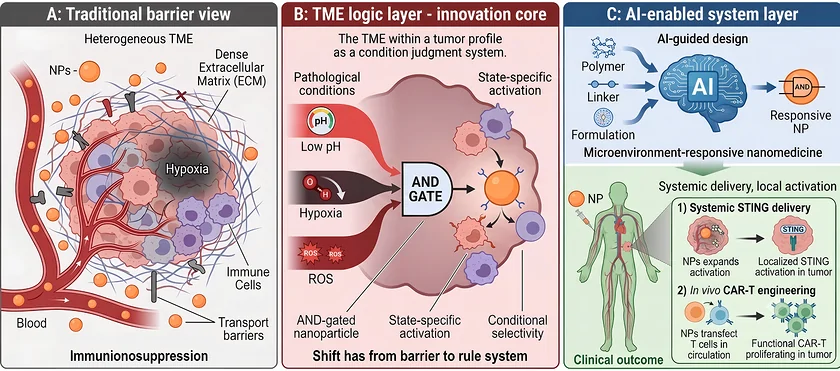
Tumor microenvironment as a conditional selectivity layer for precision nanomedicine. (A) The TME as a transport and immunosuppressive barrier. (B) Illustration of how pathological features such as acidity, hypoxia, and redox imbalance can be used as conditional inputs for locally activated nanomedicines. (C) AI‐assisted design of TME‐responsive systems. These concepts remain emerging and should be interpreted as preclinical design strategies. Figure created and/or refined using BioRender.com and ChatGPT 5.5 drawing mode; final scientific content, labels, and layout were reviewed and edited by the authors.

Effective tumor immunotherapy is constrained by two interrelated barriers: the heterogeneous immunogenicity of tumor cells and the dominance of immunosuppressive signaling within the TME [144, 145, 146]. The TME is also a highly dynamic and heterogeneous biological system that critically shapes the delivery, distribution, and therapeutic performance of cancer nanomedicines [147]. However, it should not be viewed solely as a barrier to transport. Rather, because the TME constitutes the very site at which tumor growth, immune evasion and therapeutic response unfold, it is more appropriately regarded as a key substrate for therapeutic selectivity (Figure 6A).

This distinction becomes particularly important in metastatic disease. In disseminated cancers, where lesions are spatially diffuse and biologically heterogeneous, the prospect of achieving reliable site‐by‐site targeting is inherently limited [148]. Under these conditions, nanomedicine may need to shift from site‐specific delivery toward state‐specific activation, exploiting pathological features shared across metastatic niches rather than attempting to localize each lesion individually (Figure 6B). Recent work exemplifies this concept through an AND‐gated nanoparticle design that enables systemic administration but restricts immune activation to tumor regions in which acidic pH and hypoxia coexist [149]. Such strategies suggest that the TME can be leveraged not merely as an obstacle to overcome, but as a pathological logic layer that confers conditional selectivity [150].

Within this emerging framework, AI can contribute at a level beyond empirical materials screening. By linking polymer composition, linker chemistry, and formulation parameters to microenvironment‐responsive behavior, AI‐guided workflows could help identify nanomaterials that respond to pH, redox imbalance, or hypoxia while preserving systemic stability and acceptable safety margins (Figure 6C). In practical terms, this raises the possibility that immunostimulatory agents currently restricted by systemic toxicity, such as STING agonists that are often deployed locally, could be reformulated as systemically administered but conditionally activated immunomodulators [149].

A related opportunity is emerging in in vivo CAR‐T therapy. Compared with ex vivo CAR‐T manufacturing, in vivo approaches seek to generate CAR‐T cells directly within the patient, thereby bypassing complex cell‐processing workflows, shortening time to treatment and potentially broadening access [69]. Some strategies may also reduce dependence on chemotherapy‐based lymphodepletion. Yet the beneficial functions of lymphodepletion should not be overlooked. Studies have shown that lymphodepletion enhances adoptive T cell therapy not only by reducing competition for homeostatic γc cytokines such as IL‐7 and IL‐15, but also by impairing regulatory T cell‐mediated suppression and promoting antigen‐presenting‐cell activation in the tumor context [151]. In this setting, AI could help identify the missing biological components required to reconstruct a permissive immune ecosystem in vivo, including cytokine cues, innate immune modulators, and combinatorial response modifiers. Such insight could support co‐delivery strategies that functionally recapitulate the beneficial immunological consequences of lymphodepletion without reproducing its systemic toxicity (Figure 6C).

More broadly, AI may be applied not only to engineer TME‐responsive nanomedicines but also to model the TME itself. Traditional descriptions of the TME often rely on static or reductionist features, such as vascular density or immune‐cell abundance, which fail to capture the spatiotemporal variability governing nanoparticle transport and treatment response. By contrast, AI offers a framework for treating the TME as a dynamic, learnable system (Figure 6C). Through integration of multimodal inputs, including imaging, histopathology, molecular profiling, and nanoparticle behavior, AI models may infer latent microenvironmental states and track how these states evolve under therapeutic or pathological perturbation [152]. From a nanomedicine perspective, such dynamic modeling could provide a mechanistic bridge between microenvironmental context and delivery outcomes, thereby enabling more informed prediction of vascular access, immune clearance, intracellular delivery, therapeutic windows, and downstream pharmacokinetic and pharmacodynamic behavior.

Taken together, this perspective reframes the TME from a passive impediment to a programmable source of therapeutic selectivity. For metastatic cancer in particular, the ability to move beyond lesion‐specific targeting toward pathology‐guided activation may prove essential for achieving systemic administration with local precision.

### 3.4 Predicting Biodistribution and Delivery Efficiency

The therapeutic efficacy of nanomedicines is critically shaped by their delivery efficiency and spatiotemporal distribution across tissues, yet predicting these behaviors remains a major bottleneck in preclinical development. AI‐driven modeling approaches are increasingly applied to integrate physicochemical descriptors with biological context, enabling data‐driven simulation and prediction of nanoparticle transport dynamics and biodistribution both in vitro and in vivo.

A subset of recent studies has approached nanomedicine performance primarily through the lens of delivery efficiency, treating tumor targeting or therapeutic effectiveness as the direct prediction objective. In these frameworks, ML models are trained to map nanoparticle design features, formulation parameters, or carrier attributes to quantitative measures of delivery success, effectively collapsing complex in vivo transport processes into tractable surrogate endpoints [18, 153, 154]. Such approaches enable rapid prescreening of nanoparticle designs and facilitate data‐driven prioritization without requiring exhaustive animal experimentation. Although delivery efficiency in these models often implicitly reflects underlying biodistribution patterns, biological transport processes are not explicitly resolved. Instead, efficiency is learned as an emergent outcome shaped by multiple hidden variables, offering a pragmatic but largely phenomenological route to performance prediction in cancer nanomedicine.

Complementary to efficiency‐focused prediction, a larger body of work has leveraged AI to model, infer, or reconstruct nanoparticle biodistribution across tissues and within tumors, thereby providing a mechanistic substrate from which delivery efficiency can be interpreted. These studies employ ML and hybrid modeling strategies to predict organ‐level accumulation, tumor exposure, or intratumoral spatial distribution based on physicochemical properties, microenvironmental features, or physiological transport parameters. By explicitly characterizing where nanoparticles travel and accumulate, such models capture the spatial constraints that ultimately govern therapeutic access [137, 154, 155]. Recent advances further extend this paradigm by combining deep learning with high‐resolution imaging to map nanocarrier distribution at single‐cell resolution across whole organisms, establishing empirical ground truth for in vivo delivery behavior [156]. Collectively, these efforts position biodistribution modeling not merely as an endpoint assessment, but as a critical intermediate layer linking nano–bio interactions to delivery efficiency and downstream pharmacological outcomes.

Despite encouraging progress, current AI‐based approaches for predicting nanomedicine delivery efficiency and biodistribution remain constrained by several fundamental limitations. Most existing models rely on static or endpoint measurements, treating delivery as a fixed outcome rather than a dynamic, time‐dependent process governed by evolving biological states [157]. As a result, temporal aspects of transport, clearance, and redistribution are often ignored or implicitly absorbed into surrogate labels. In addition, many models are trained on relatively narrow experimental domains, limiting their ability to generalize across tumor types, disease stages, or nanoparticle platforms. The biological interpretability of learned predictors also remains limited, as efficiency is frequently inferred without explicit representation of underlying transport mechanisms or microenvironmental constraints. Recent studies increasingly acknowledge these challenges and point toward future frameworks that integrate richer physiological context, spatially resolved data, and mechanistic priors, including hybrid PBPK‐ML models and multimodal training strategies [158]. Looking forward, advancing from phenomenological prediction toward unified, state‐aware modeling of biodistribution and delivery efficiency will require AI systems capable of learning transferable representations across scales, bridging nano–bio interactions, tissue‐level transport, and downstream pharmacological outcomes.

## 4 Why Nanomedicine Fails: System‐Level Constraints and the Role of AI

Nanomedicine has long been regarded as a promising strategy to overcome fundamental limitations of conventional cancer therapies, offering opportunities to modulate drug distribution, exposure, and therapeutic index through rational carrier design [159]. However, despite decades of innovation, its clinical impact remains limited and inconsistent [5]. This gap cannot be fully explained by insufficient materials innovation alone, but reflects a deeper mismatch between nanoscale engineering strategies and the multiscale complexity of biological and clinical systems [160].

At its core, nanomedicine performance emerges from the interaction between material properties, biological environments, and clinical context, rather than from any single design parameter. Conventional development paradigms, which optimize formulation components in isolation, are therefore inherently insufficient. As a result, improvements achieved at the level of carrier design often fail to translate into meaningful clinical benefit.

**FIGURE 7:**
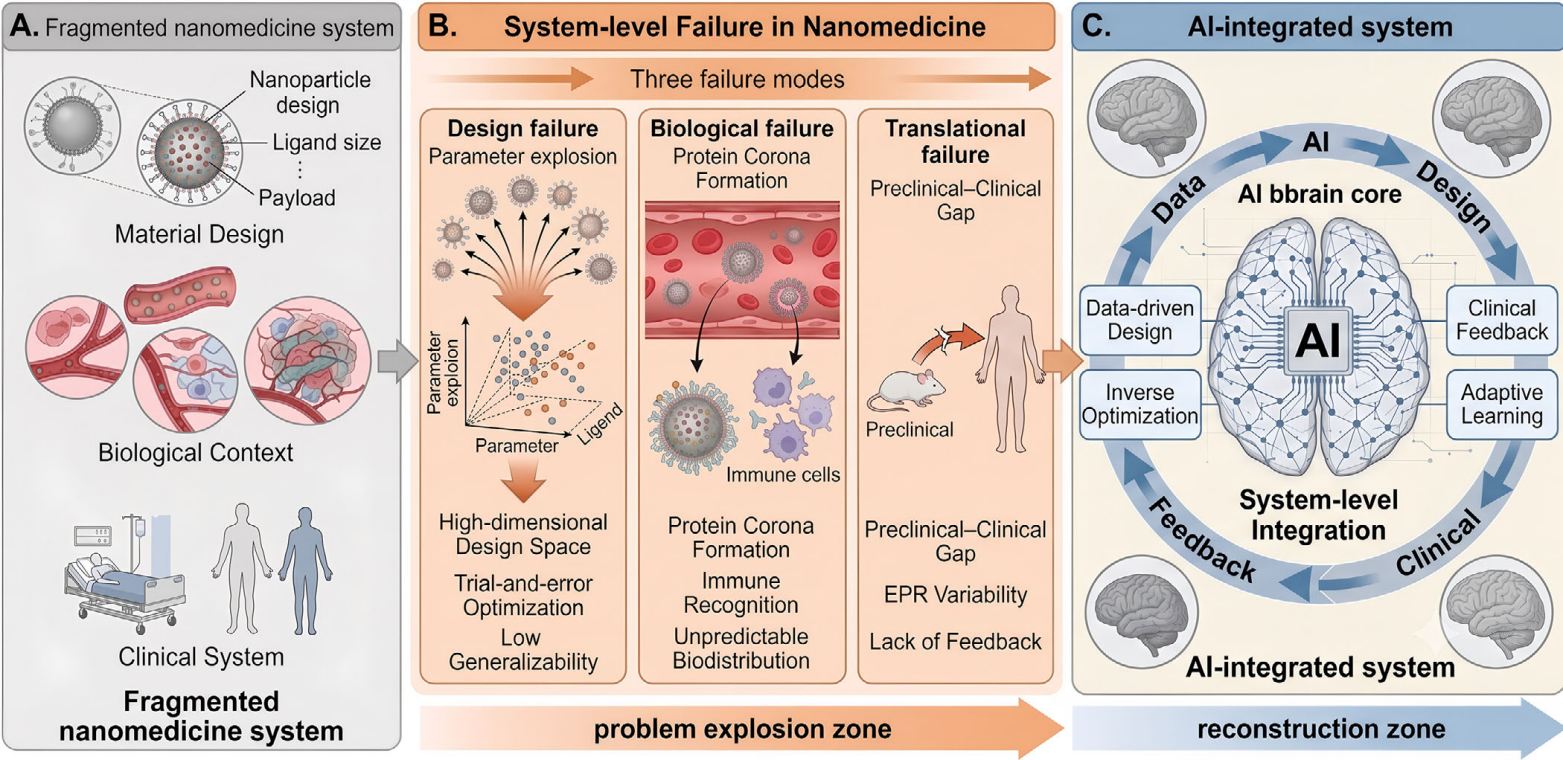
From fragmented optimization to integrated nanomedicine development. Conventional pipelines often separate material design, biological evaluation, and clinical deployment, contributing to design, biological, and translational failure modes. AI can connect these domains through structured data integration, predictive modeling, and feedback‐informed refinement, supporting more coherent development across design, delivery, and clinical evaluation. Figure created and/or refined using BioRender.com and ChatGPT 5.5 drawing mode; final scientific content, labels, and layout were reviewed and edited by the authors.

From a system‐level perspective, these limitations arise from three interconnected failure modes: (i) high‐dimensional design without integrative optimization, (ii) unpredictable nano–bio interactions in vivo, and (iii) discontinuity between preclinical development and clinical deployment (Figure 7). These constraints reinforce each other, forming a translational bottleneck that cannot be resolved through incremental material innovation alone.

### 4.1 Design Failure: High‐Dimensional Formulation Without Integration

Nanoparticle‐based drug delivery systems provide a versatile platform capable of encapsulating diverse therapeutic payloads, improving pharmacokinetics, and enabling spatiotemporal control of drug release. They also support combination strategies through co‐delivery of synergistic agents and integration with immunotherapy or gene‐based approaches. However, these advantages are typically realized under controlled experimental conditions and do not directly translate into consistent in vivo performance. A central limitation lies in the high‐dimensional nature of nanomedicine design, which involves multiple interdependent variables, including material composition, particle size, surface chemistry, targeting ligands, and payload properties [160, 161, 162].

Conventional development strategies rely on iterative and low‐throughput experimentation, exploring this design space through fragmented trial‐and‐error workflows (Figure 7). Such approaches are inefficient and often yield locally optimized formulations that perform well in specific experimental settings but lack robustness across biological contexts. In addition, preclinical datasets are frequently sparse, heterogeneous, and poorly standardized, limiting their reuse and preventing accumulation of transferable design principles. As a result, knowledge remains localized rather than generalizable, and promising candidates may be overlooked while suboptimal ones advance [5, 163, 164, 165].

From a systems perspective, this bottleneck reflects not only insufficient exploration of design space but also the absence of integrative frameworks linking physicochemical properties to biological function.

### 4.2 Biological Failure: Unpredictable Nano–Bio Interactions In Vivo

Beyond design complexity, a major limitation of nanomedicine lies in the unpredictable nature of nanoparticle interactions with biological systems (Figure 7). Following systemic administration, nanoparticles encounter complex and dynamic environments shaped by protein adsorption, immune recognition, vascular transport, and tissue‐specific barriers [166, 167]. These processes collectively determine biodistribution, cellular uptake, and therapeutic efficacy, yet remain difficult to predict from in vitro assays or simplified models. For example, the enhanced permeability and retention (EPR) effect, widely considered a key mechanism for tumor targeting, is highly variable in clinical settings and often fails to produce consistent therapeutic benefit [63, 168, 169]. Similarly, immune interactions, including sequestration by monocytes and neutrophils, complement activation, and protein corona‐mediated redefinition of nanoparticle identity, introduce additional layers of context‐dependent variability [170, 171, 172, 173]. These observations indicate that nanomedicine performance cannot be inferred solely from static material properties, but instead emerges from dynamic interactions between nanoparticles and biological systems. The lack of predictive frameworks for these interactions remains a major barrier to rational design.

### 4.3 Translational Failure: Discontinuity Between Preclinical and Clinical Systems

A further critical limitation arises from the discontinuity between preclinical optimization and clinical performance (Figure 7). Although many nanomedicine formulations demonstrate promising efficacy in vitro and in animal models, these results frequently fail to translate into meaningful clinical benefit [6, 174, 175, 176]. This discrepancy reflects fundamental differences in physiology, immune environment, and tumor heterogeneity between experimental systems and human patients [177]. Delivery mechanisms such as the EPR effect, which are often central to preclinical success, are poorly reproducible in clinical contexts [63, 168, 169]. In addition, the absence of standardized frameworks for pharmacokinetic modeling, patient stratification, and quality control further complicates clinical translation and regulatory evaluation [178, 179, 180]. Critically, current nanomedicine development pipelines remain largely open‐loop. Preclinical findings rarely feed back into model refinement based on clinical outcomes, and clinical data are seldom integrated into upstream design processes. As a result, knowledge accumulation remains fragmented and non‐cumulative, limiting the ability to improve system performance over time.

### 4.4 AI as an Integrative Solution to System‐Level Failure

Importantly, the role of AI in nanomedicine should not be viewed as a collection of isolated applications, such as formulation optimization or molecular design. Rather, AI represents a potential integrative framework capable of addressing system‐level fragmentation across the nanomedicine development pipeline [14, 181, 182].

By enabling structured data integration, predictive modeling, and iterative learning, AI can connect previously disconnected processes, including material design, biological evaluation, and clinical decision‐making [183, 184, 185]. This includes linking nanoparticle properties to in vivo behavior, integrating preclinical data with active learning strategies, and incorporating clinical observations into continuous model refinement (Figure 7).

Accordingly, AI can help coordinate nanomedicine development across material design, biological evaluation, and clinical use. The next section focuses on the practical requirements for this coordination, including data standards, learning workflows, clinical stratification, and governance.

## 5 Translational Control: Data, Learning Paradigms, and Clinical Deployment

Section 4 identified fragmentation across design, biology, and clinical deployment as a core reason for translational failure [182]. This section turns from that conceptual diagnosis to implementation: the data quality, learning workflows, clinical stratification strategies, and governance structures required for AI‐supported nanomedicine to become reproducible and clinically credible.

**FIGURE 8:**
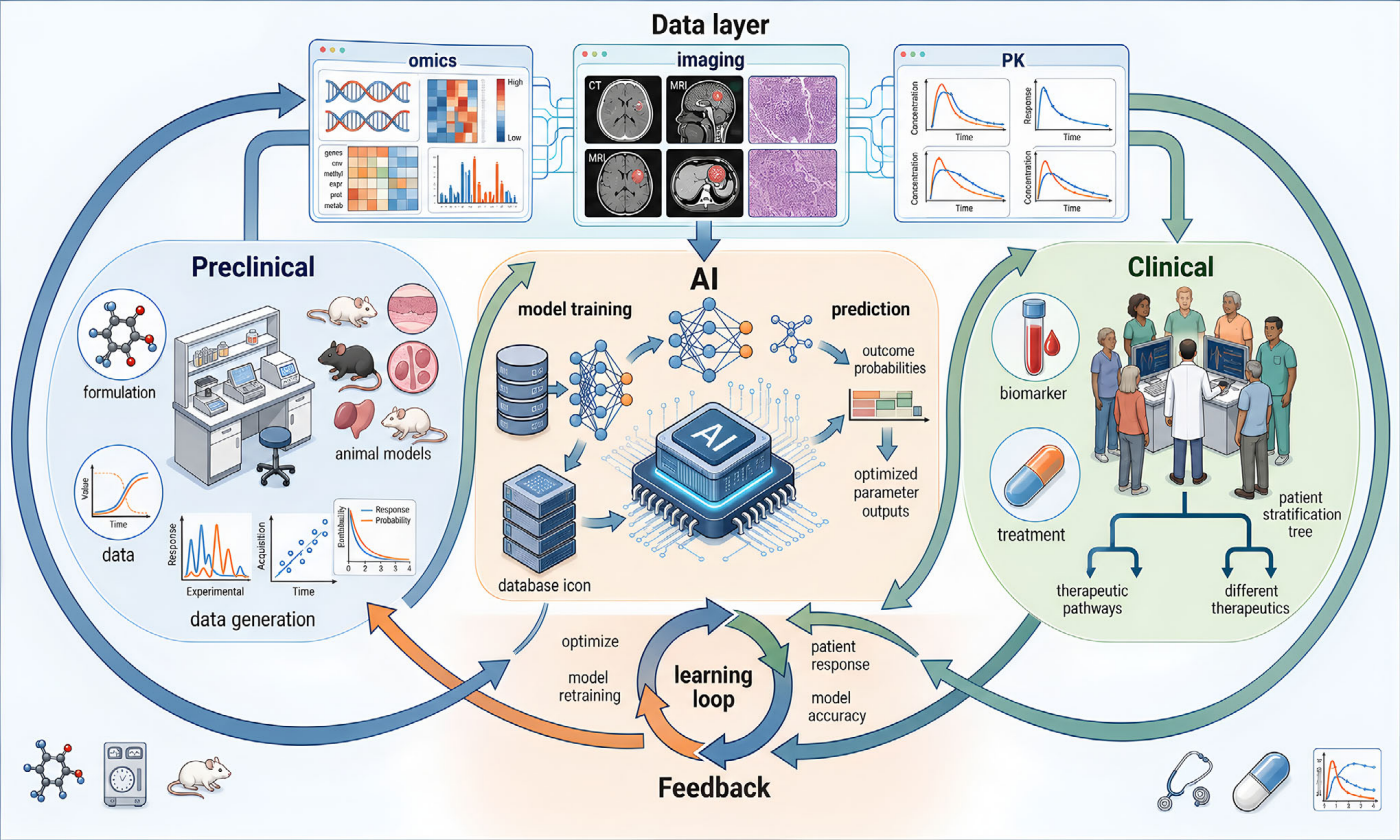
Translational framework for AI‐guided nanomedicine. Standardized data, preclinical learning workflows, nano‐enabled clinical monitoring and governance structures connect discovery‐stage optimization with clinical feedback, supporting reproducible development, patient stratification and evidence‐based therapeutic refinement. The framework is intended as a translational roadmap rather than evidence of current fully closed‐loop clinical deployment. Figure created and/or refined using BioRender.com and ChatGPT 5.5 drawing mode; final scientific content, labels, and layout were reviewed and edited by the authors.

The emphasis here is therefore operational. Rather than restating AI as an integrative concept, we examine how structured datasets, preclinical learning workflows, and clinically informed decision frameworks can convert isolated observations into cumulative and actionable knowledge [15]. Figure 8 summarizes this implementation‐oriented translational framework.

### 5.1 Data Quality, Standardization, and Interoperability

High‐quality, standardized, and interoperable data are prerequisites for effective AI in nanomedicine. Data do not simply serve as inputs but define the boundaries of what can be learned, predicted, and generalized [186]. The representational capacity of any model is therefore constrained not only by algorithmic architecture but also by the coherence, granularity, and conceptual validity of the underlying datasets.

This dependency is particularly critical in supervised learning, where predefined labels determine which aspects of nanomedicine can be rendered learnable [187]. While ML can accelerate experimentation by prioritizing informative designs and enabling high‐throughput screening, such benefits are conditional on data quality. Models trained on sparse, inconsistently annotated, or context‐dependent datasets will propagate experimental bias rather than resolve it [188].

These challenges are especially pronounced in cancer nanomedicine, where data are often generated through heterogeneous laboratory workflows [186]. Variability in protocol execution, measurement conditions, and metadata reporting introduces latent confounders that can become embedded in both features and labels, limiting model interpretability and generalizability.

Addressing this limitation requires a shift from data accumulation to data infrastructure. Key elements include standardized experimental protocols, machine‐readable metadata, interoperable ontologies, persistent identifiers, controlled vocabularies, and FAIR‐compliant data stewardship [189]. Recent nanosafety‐oriented efforts further illustrate how these principles can be operationalized through modular metadata schemas derived from existing standards and through template‐based tools for machine‐readable data reporting [190, 191]. Such approaches are particularly valuable for nanomedicine because they convert heterogeneous experimental descriptions into structured records that can be searched, compared, integrated, and reused by computational models. In practical terms, AI‐ready nanomedicine datasets should report not only final outcomes but also formulation identifiers, batch information, material source, synthesis and processing parameters, physicochemical characterization, colloidal stability, protein‐corona conditions, biological model, dose metrics, exposure duration, assay protocol, endpoint definition, negative results, and uncertainty estimates.

Several existing community resources provide useful starting points for this transition (Table 2). The MIRIBEL reporting framework defines minimum information for bio–nano experimental studies, including material characterization, biological characterization, and experimental protocols [186]. eNanoMapper provides an ontology‐based infrastructure for annotating nanomaterial composition, physicochemical properties, assay conditions, biological endpoints, and safety‐related data [192]. NanoMine offers a machine‐readable schema for polymer nanocomposite data and illustrates how material composition, processing, structure, and properties can be organized for data mining and model development [193]. Although these resources were not all designed specifically for cancer nanomedicine, they provide practical templates for transforming heterogeneous experimental records into structured, reusable, and computationally learnable datasets.

**TABLE 2:** Practical data‐infrastructure recommendations for AI‐enabled nanomedicine.

Resource or initiative	Primary function	Practical recommendation for AI‐enabled cancer nanomedicine
FAIR principles	General framework for findable, accessible, interoperable, and reusable data	Assign persistent identifiers, use machine‐readable metadata, define access rules, and report reusable data formats and licenses [189]
MIRIBEL	Minimum information reporting framework for bio–nano experimental literature	Use as a checklist for material characterization, biological characterization, assay conditions, dose metrics, and protocol details [194]
eNanoMapper	Ontology‐based infrastructure for nanomaterial safety and biological interaction data	Use controlled terms for nanomaterial type, composition, physicochemical properties, experimental model, assay endpoint, and exposure condition [192]
NanoMine	Data schema and resource for polymer nanocomposite structure–processing–property relationships	Adapt its schema‐oriented logic to organize formulation composition, processing variables, structural descriptors, and functional outcomes for model training [193]
Implementation priority	Translation‐oriented data governance	Combine standardized metadata, ontology mapping, raw‐data deposition, quality‐control metrics, negative‐result reporting, and batch‐level traceability before model training [189, 192, 194]
Modular metadata schema for nanosafety	Standard‐derived metadata framework for nanosafety research data	Use modular fields to describe material identity, synthesis, characterization, exposure context, biological model, assay conditions, endpoint definitions, and provenance [190]
Template wizard for machine‐readable reporting	User‐facing tool for co‐creating structured reporting templates	Use template‐guided reporting to harmonize experimental metadata before data deposition, thereby improving machine readability, cross‐study comparison, and AI model training [191]

From a translational perspective, reliable AI is inseparable from reliable data. Progress therefore depends not only on improved algorithms but also on the establishment of structured, transparent, and learning‐compatible data regimes that allow models to be reproduced, compared, audited, and transferred across experimental and clinical settings.

### 5.2 Preclinical Closed Loops and Self‐Driving Laboratories

If data quality determines whether nanomedicine can be learned, then closed‐loop development determines how such data are generated. In preclinical nanomedicine, closed‐loop optimization reframes development as an iterative design‐build‐test‐learn process, in which AI models guide experimental decisions and are continuously updated with new observations [195]. This paradigm shifts nanomedicine research from hypothesis‐driven experimentation toward data‐driven optimization. Rather than exploring formulation space through fragmented trial‐and‐error, closed‐loop systems prioritize informative experiments, thereby improving efficiency while enhancing data structure and consistency.

Self‐driving laboratories represent the most advanced implementation of this concept, integrating automated synthesis, robotic handling, high‐throughput characterization, and machine‐learning‐guided decision engines into unified experimental platforms [196, 197, 198]. These systems are particularly well suited to nanomedicine, where design spaces are high‐dimensional and experimentally costly [199]. By coupling active learning with automation, they enable efficient navigation of formulation landscapes while reducing operator‐dependent variability [199, 200, 201]. Importantly, translational value does not depend on full automation. Partial closed‐loop systems, combining AI‐guided decision‐making with human‐supervised experimentation, can already provide substantial gains in reproducibility, throughput, and information yield. Closed‐loop development should therefore be viewed as a continuum rather than a binary threshold.

Despite these advances, current systems remain largely laboratory‐bound. Challenges including high infrastructure costs, limited interoperability, and poor cross‐domain generalization constrain their scalability [202]. Moreover, early‐stage optimization often neglects manufacturability, leading to formulations that perform well in controlled settings but fail during scale‐up.

The long‐term objective is not simply automation, but the construction of standardized, interoperable preclinical learning systems that integrate discovery with manufacturability, reproducibility, and translational feasibility. In this framework, closed loops evolve from experimental accelerators into foundational infrastructure for clinically robust nanomedicine.

### 5.3 Clinical Learning Loops and Precision Stratification

The logic of closed‐loop learning extends beyond preclinical development into clinical deployment. Clinical learning loops provide a framework for integrating longitudinal patient data, AI‐driven interpretation, and adaptive therapeutic decision‐making into a continuous optimization process [203]. In conventional clinical workflows, data are often treated as static endpoints analyzed retrospectively. In contrast, a clinical loop treats patient data as dynamic inputs that inform ongoing therapeutic adjustment [204]. This transformation is particularly relevant for nanomedicine, where treatment performance is highly dependent on patient‐specific biological context.

Nanotechnology plays a central role in enabling this framework by enhancing the sensing layer of clinical systems. Nano‐enabled diagnostics can amplify weak signals, capture rare biomarkers, and generate high‐dimensional representations of patient state across imaging, molecular profiling, and circulating biomarkers. These multimodal datasets provide a rich substrate for AI‐driven inference, as summarized in Table 3.

**TABLE 3:** Representative AI‐enabled nanoplatforms for cancer diagnosis, screening. and liquid biopsy.

Clinical application	AI model	Analysis task	Nanoplatform/assay	Key finding/clinical value
Pan‐cancer screening, prognosis and biomarker detection	ML/DL	Predictive modeling	2D‐material biosensing platforms	Portable, low‐cost and highly sensitive cancer detection/monitoring potential [214]
Peptide biomarker detection in biofluids	PCA + semi‐supervised learning	Nanopore signal classification	Protein nanopore single‐molecule sensor	Resolved closely related serum peptide biomarkers and conformers [215]
EV heterogeneity and structural profiling	Neural network	Image segmentation and quantification	Cryo‐TEM‐based EV analysis platform	High‐throughput mapping of stable EV structural heterogeneity [216]
mtDNA molecular diagnostics and biomarker quantification	SVM	Nanopore classification and quantification	Solid‐state nanopore single‐molecule sensor	Amplification‐free, highly specific mtDNA quantification from low‐abundance samples [217]
Exosome source identification and early cancer detection support	ANN	Raman classification and deconvolution	Label‐free SERS exosome platform	Separated cancer‐derived from healthy exosomes; estimated tumor‐derived fraction in mixtures [218]
Liquid‐biopsy screening of cancer and precancer	ML	RNA feature selection and panel building	Multichannel nanofluidic exosome isolation and RNA‐analysis platform	Discriminated healthy, precancer, and cancer states; supported pancreatic cancer detection [206]
Cancer‐associated VTE risk assessment	ML	EV–clinical‐data integration	TiO_2_ nanoflower procoagulant EV barcode assay	High‐sensitivity/high‐specificity VTE risk stratification [219]
Multicancer pathology and intraoperative guidance	ML	Fluorescence‐image classification	Survivin‐antibody‐functionalized perovskite nanocrystal probe	Rapid tumor/adjacent‐normal discrimination and organ‐of‐origin prediction [220]
Early bladder cancer detection and progression assessment	PCA/PLS‐DA	Urine spectral classification	Au‐coated ZnO nanoporous SERS chip	Single‐drop urine test distinguished cancer‐free, early, and polyp‐stage bladder cancer [221]
Minimally invasive GBM diagnosis and brain tumor discrimination	ML	CIV profiling and classification	GBM ImmunoProfiler SERS circulating immune‐vesicle platform	Accurate GBM detection from minimal peripheral blood; differentiated brain tumor types [222]
Brain cancer liquid biopsy, deep subtyping and monitoring	DL	Serum Raman classification and localization	3D nanosensor SERS serum platform	Distinguished primary vs. secondary brain tumors and predicted lesion location [223]
Brain metastasis liquid biopsy and origin prediction	ML	EV profiling and classification	Brain nanoMET SERS exosome platform	Separated metastatic from primary brain tumors and predicted tissue of origin [224]
Breast cancer screening and classification	DL	Spectral‐image classification	Ag‐nanoparticle‐enhanced serum SERS platform	Automated high‐accuracy discrimination of patients vs. healthy controls [225]
Breast cancer liquid biopsy and stage discrimination	ML	Multiplex protein‐pattern analysis	Au SERS nanotags plus sEV‐capture platform	Distinguished healthy, in situ, early and metastatic breast cancer [226]
Breast cancer liquid biopsy and heterogeneity stratification	ML	Single‐cell spectral profiling	Immuno Nano Sensor for immune‐cell metabolic profiling	Distinguished cancer vs. healthy, primary vs. metastatic disease, and ER/PR status [227]
Early breast cancer screening	DL	SERS classification	3D plasmonic‐cluster plasma SERS platform	High‐accuracy plasma molecular‐fingerprint screening [228]
Noninvasive plasma screening for breast cancer	ML	Plasma Raman classification	Microcapillary‐confined Au nanocluster SERS platform	Rapid plasma screening without sample pretreatment [229]
Noninvasive colorectal cancer diagnosis	ML	Methylation‐related spectral profiling	Raman‐based NK‐cell DNA assay	CRC discrimination via NK‐cell DNA methylation fingerprints [230]
Early colorectal cancer screening and differential diagnosis	XGBoost	CTC/RNA integration and risk scoring	Dual‐antibody antifouling hydrogel‐coated magnetic nanoparticles for CTC capture	Distinguished CRC, polyps, and adenomas; 91.0% diagnostic accuracy [231]
Early gastric cancer diagnosis and disease monitoring	ML	Exosomal metabolomics	Aptamer‐coupled Au‐modified polymorphic‐carbon exosome‐capture platform	Separated early gastric cancer from healthy controls; identified monitoring‐related metabolites [232]
Noninvasive gastric cancer screening	ML	Serum Raman classification	Ag@SiO_2_ core‐shell SHINERS serum platform	Rapid discrimination of gastric cancer from low‐volume serum [205]
Noninvasive gastric cancer screening	ML	sEV Raman classification	Au nanopyramid‐array SERS exosome platform	Detected gastric cancer from tissue‐, blood‐, and saliva‐derived sEVs [233]
Hepatocellular carcinoma liquid biopsy and differential diagnosis	ML	Tumor‐EV subpopulation analysis	DCR‐IEVN tumor‐derived EV nanoencapsulation platform	Distinguished HCC, cirrhosis, and healthy controls in a simplified workflow [234]
Lung cancer diagnosis and subtype classification	ML	Multi‐miRNA profiling and classification	Size‐coded hydrogel‐bead liquid‐biopsy platform	Improved diagnostic accuracy; ∼80% subtype‐classification accuracy [235]
Noninvasive lung cancer diagnosis and stratification	ML	T‐cell SERS profiling	T‐sense nanosensor and SERS immune‐profiling platform	Distinguished primary vs. metastatic lung cancer from peripheral T cells [236]
Noninvasive lung cancer screening and benign/malignant discrimination	Logistic regression	Salivary Raman classification	3D‐PHP paper‐based SERS saliva platform	Rapid point‐of‐care screening with benign/malignant separation potential [237]
Lung liquid biopsy and cytopathology support	DL	Rare‐cell detection and slide review	PERFECT micro‐/nanotech liquid‐biopsy membrane platform	Unbiased BALF rare‐cell detection; outperformed manual review [238]
Early ovarian cancer detection and stage classification	ML	Multiplex‐signal modeling	MoS_2_ composite‐film SERS exosome platform	Separated healthy controls and stage‐specific ovarian cancer [209]
Noninvasive urinary diagnosis of prostate and pancreatic cancers	DL	Label‐free urine Raman classification	3D‐PCN paper‐based SERS urine strip	High sensitivity and specificity with point‐of‐care potential [239]
Noninvasive thyroid cancer screening and differential diagnosis	ML	Urinary metabolomic classification	Au‐doped ZrMOF metabolic‐fingerprinting platform	Distinguished thyroid cancer from low‐risk nodules; reduced unnecessary invasive work‐up [240]
Noninvasive thyroid cancer diagnosis	DL	Serum Raman classification	3D Au nanocluster SERS serum platform	Biomarker‐free, metabolic‐fingerprint‐based auxiliary diagnosis [241]
Follicular lymphoma risk stratification and progression prediction	Unsupervised ML	Proteomic stratification	Nano‐LC‐MS/MS proteomic profiling	Identified high‐risk patients at diagnosis and candidate prognostic proteins [242]

Abbreviations: ANN, artificial neural network; BALF, bronchoalveolar lavage fluid; CIV, circulating immune vesicle; CRC, colorectal cancer; DL, deep learning; EV, extracellular vesicle; GBM, glioblastoma; HCC, hepatocellular carcinoma; PCA, principal component analysis; PLS‐DA, partial least squares discriminant analysis; SERS, surface‐enhanced Raman scattering; sEV, small extracellular vesicle; SHINERS, shell‐isolated nanoparticle‐enhanced Raman spectroscopy; SVM, support vector machine; VTE, venous thromboembolism.

This observational role can be understood across three complementary diagnostic strata. At the imaging level, nano‐enabled amplification of optical and physical signals, for example through surface‐enhanced Raman spectroscopy, shell‐isolated nanoparticle‐enhanced Raman platforms or nanoscale mechanical imaging, produces high‐dimensional phenotypic fingerprints that AI can decode into diagnostic classes and disease states [205]. At the omics level, nanoplatforms act as concentrators and translators of nucleic acid and proteomic information, enabling the reconstruction of tumor‐associated molecular patterns that are often difficult to resolve using standard assays alone [206]. At the systemic level, nano‐assisted liquid biopsy and circulating biomarker platforms extend this logic beyond the local tumor site, capturing extracellular vesicles, blood‐based signatures, and other organism‐wide indicators of malignancy and treatment response [207, 208, 209]. Taken together, these layers do more than improve diagnosis: they supply the longitudinal, multimodal, and clinically updateable variables needed to support a true clinical learning architecture.

By integrating these data, AI can construct dynamic and probabilistic representations of disease progression, treatment response, and toxicity risk [62, 210, 211]. This enables adaptive patient stratification beyond single biomarkers and supports real‐time treatment optimization.

In this context, patient stratification becomes the operational bridge between nano‐enabled diagnostics and precision therapy. Multimodal integration enables more accurate subgroup identification, dynamic risk assessment, and improved matching of patients to nanomedicine‐based interventions [212, 213].

### 5.4 Interpretability, Causal Reasoning, Validation and Risk‐Based Deployment

As AI systems move toward clinical deployment, predictive accuracy alone is insufficient. Models intended to support formulation decisions, manufacturing control, or clinical intervention must be interpretable, prospectively validated, and evaluated within a clearly defined context of use [243]. In nanomedicine, this requirement is particularly important because model outputs may influence patient selection, dose strategy, batch release, toxicity prediction, or regulatory decision‐making.

Model interpretability can help connect statistical predictions to mechanistic nano–bio understanding. Feature‐attribution methods such as SHAP can identify which formulation variables, corona components, imaging features, or patient‐level covariates contribute most strongly to a prediction, whereas attention‐based architectures may highlight influential molecular or imaging regions. These tools should not be treated as proof of mechanism, but they can generate experimentally testable hypotheses and improve transparency for regulators, clinicians, and formulation scientists [244].

A further step is to move from correlation toward causation. Many AI models learn associations between nanoparticle descriptors and biological outcomes, but these associations may reflect confounding by assay conditions, tumor model, dose, route of administration, or laboratory workflow. Causal inference frameworks, including directed acyclic graphs, counterfactual reasoning, and perturbation‐based validation, can help distinguish predictive correlates from causal drivers of delivery, toxicity, or therapeutic response [245]. In practice, this requires prospective validation, external datasets, mechanistic assays, and perturbation experiments that test whether modifying a predicted driver changes the biological outcome.

Regulatory and translational deployment should therefore integrate interpretability, causal plausibility, batch‐to‐batch reproducibility, toxicity prediction, and patient stratification into a single risk‐based validation strategy [246]. Data governance frameworks are equally critical to enable secure, ethical, and interoperable data sharing across institutions. Given the potential for bias, performance drift, and limited generalizability, AI‐enabled nanomedicine should be monitored and recalibrated throughout its lifecycle rather than validated only once. Ultimately, the success of AI‐enabled nanomedicine depends not on removing humans from the loop, but on precisely defining their role in guiding, validating, and governing intelligent systems.

## 6 Future Directions for AI‐Powered Cancer Nanomedicines

Despite the progress reviewed above, AI‐enabled cancer nanomedicine remains constrained by task‐specific supervised models, endpoint‐oriented prediction, and fragmented validation across experimental, biological, and clinical settings [194, 247, 248]. Future work should therefore address three unresolved needs: transferable learning frameworks [249, 250], dynamic multiscale models [251], and patient‐aware therapeutic strategies [252, 253]. These directions extend the translational framework discussed in Section 5 by defining what next‐generation models must learn, how they should represent biological complexity, and how they can support clinically meaningful decisions.

### 6.1 Toward More Unified Learning Frameworks

The priority is to move beyond models optimized for narrow tasks toward representations that capture shared principles of nanoparticle behavior across materials, biological contexts, and therapeutic settings. Active targeting, payload optimization, biodistribution modeling, and translational control all point to the same need: AI systems that can transfer knowledge across related problems rather than relearning each problem from scratch.

Supervised learning, which learns mappings from inputs to outputs using labeled examples, will remain indispensable whenever the objective is a predefined and operationally meaningful endpoint, such as a critical quality attribute, a toxicity threshold, or a clinically interpretable response variable [254]. Yet its strengths also define its boundaries [255]. Because supervised models are tightly coupled to the labels used to train them, they inevitably inherit the sparsity, noise, semantic inconsistency, and experimental context‐dependence of those labels [256]. In cancer nanomedicine, where many endpoints are generated through heterogeneous assays, local workflows, or surrogate readouts that only partially reflect patient‐relevant biology, this dependence can make even accurate models brittle under domain shift and difficult to translate into mechanistic insight [257, 258]. The challenge, therefore, is not to abandon supervision, but to move beyond exclusively label‐bound learning.

Unsupervised and self‐supervised learning are important because they can extract transferable structure from large, heterogeneous, and only partially annotated datasets, thereby reducing dependence on fragile endpoints [259, 260]. In principle, such approaches could help align physicochemical descriptors, protein‐corona profiles, manufacturing metadata, imaging signatures, biodistribution maps, and clinical observations into more coherent latent representations. If successful, these representations would make downstream models more data‐efficient, more portable across nanoparticle classes, and better able to connect design variables with biological function without requiring each new task to be learned independently [261]. Recent work illustrates this direction in a concrete way; Xu et al. reported LUMI‐lab, a foundation‐model‐driven self‐driving platform for ionizable lipid discovery that combines large‐scale pretraining, active learning, and automated experimentation to address sparse historical data [201]. The broader goal is not unsupervised learning for its own sake, but the emergence of learning architectures that can accumulate knowledge across tasks, contexts, and scales while remaining credible enough to guide design and translation.

### 6.2 Toward Dynamic and Multiscale Modeling

A second future direction emerges directly from a limitation highlighted throughout the preceding sections: many models still rely on endpoint‐style or narrowly defined labels and therefore remain only weakly dynamic. They are typically trained on endpoint measurements, snapshot phenotypes, or narrowly defined surrogate labels, and therefore struggle to capture how nanoparticle behavior evolves over time and across biological scales. Yet nanomedicine performance is not determined by a single state. It emerges from a cascade of coupled processes, spanning material properties, nano–bio interactions, vascular transport, tissue distribution, microenvironmental adaptation, and downstream therapeutic response. As a result, models that remain fixed at one timepoint or scale are unlikely to provide a sufficiently faithful account of real in vivo behavior.

This limitation points to the need for more dynamic and multiscale frameworks. Rather than treating delivery, biodistribution, or efficacy as isolated outputs, future AI systems may need to connect events across levels of organization, from particle–scale interactions and local flow physics to organ‐level pharmacokinetics and patient‐level treatment dynamics. Recent work already illustrates the feasibility of this direction. For example, machine‐learning‐enabled multiscale models have begun to couple nanoparticle margination in microvasculature with PBPK descriptions of whole‐body biodistribution, showing how transport across scales can be represented within a single predictive framework [262]. More broadly, such approaches suggest that the next advance may come not from improving one predictor at a time, but from linking previously separate models into coordinated representations of nanomedicine behavior [9].

Within this broader trajectory, digital twins represent a particularly ambitious extension. In oncology, digital twins are increasingly conceived as virtual representations that evolve with incoming data and can be used to simulate treatment response, explore individualized trajectories, and support adaptive decision‐making [263, 264, 265]. For cancer nanomedicine, the long‐term attraction of this concept lies in its potential to integrate formulation properties, biological transport, patient‐specific molecular information, and longitudinal clinical observations into a continuously updated model of therapeutic behavior. Such systems remain largely aspirational, and their realization will require far richer data, stronger mechanistic priors, and closer integration between experimentation, modeling, and clinical monitoring [266]. Even so, the conceptual shift is already important: the field may need to move beyond static prediction toward living models that update as nanomedicine moves through design, delivery, and patient response.

### 6.3 Toward Adaptive and Patient‐Aware Nanomedicine

A third future direction follows from the same logic: nanomedicine development must become more attentive to patient‐specific biological context. Current pipelines often assume that once a carrier and payload are optimized, the resulting formulation can be broadly deployed with limited adjustment beyond dose or indication. Yet performance is strongly shaped by tumor microenvironmental state, immune interaction, biodistribution dynamics, clinical heterogeneity, and treatment history [267, 268, 269]. The central challenge is therefore not only how to design better nanoparticles, but how to match them to biologically defined patient states.

Nano‐enabled diagnostics, longitudinal clinical data, and AI‐driven stratification provide a path beyond one‐size‐fits‐all deployment [270]. Future frameworks may increasingly match carrier architectures, targeting strategies, or payload combinations to delivery phenotypes, immune states, and risk profiles, then refine these assignments as new imaging, molecular, or circulating biomarker data become available. This would align nanomedicine more closely with precision oncology by improving both initial patient selection and ongoing therapeutic adjustment.

Such adaptation must remain evidence‐based, auditable, and proportionate to clinical risk. The aim is not infinitely individualized formulations for every patient, but learning systems that distinguish robust design features from context‐sensitive variables that can be tuned in response to meaningful biological variation [271]. Under this view, AI supports not only pre‐use optimization but also the responsible adjustment of nanomedicine strategies throughout clinical deployment [265].

## 7 Conclusion

Cancer nanomedicine has long been envisioned as a platform for improving therapeutic precision, yet its clinical impact has remained limited by the complexity and unpredictability of nano–bio interactions. This challenge is not solely a matter of materials design, but reflects a deeper disconnect between nanoscale engineering and biological systems across multiple scales.

AI offers a conceptual and practical route to address this gap by connecting material design, nano–bio interactions and clinical evidence within a more coherent development framework. Its value lies in making relationships among formulation variables, biological interfaces and response patterns more explicit, testable, and reusable.

This does not mean that every nanomedicine must become fully automated or individually customized. Rather, AI can help identify which design variables are robust, which biological contexts are decisive, and which patient groups are most likely to benefit.

Despite these advances, significant challenges remain. Data heterogeneity, limited standardization, and domain shift between experimental and clinical settings constrain model generalizability. In addition, the complexity of biological systems and the need for robust validation impose stringent requirements on model interpretability and reliability. Addressing these challenges will require not only methodological innovation, but also coordinated efforts in data governance, infrastructure development, and interdisciplinary collaboration.

The field's next task is therefore to pair model innovation with data standards, mechanistic validation, and clinically meaningful evaluation. Progress will depend on frameworks that are not only predictive, but also interpretable, reproducible, and compatible with real translational constraints. If these conditions are met, AI could help move cancer nanomedicine from empirical formulation toward a more predictive and precision‐oriented therapeutic discipline in oncology.

## Author Contributions

L.S.B. and L.Z. performed conceptualization, methodology, and investigation, wrote the original draft, and edited the final manuscript. W.C.Y., S.H.X., and Z.R.J. wrote the original draft and edited the manuscript. L.S.B. and S.X.R. provided supervision. All authors have read and approved the final manuscript.

## Funding

This study was supported by the National Key Research and Development Program of China (2023YFC3403200), the National Natural Science Foundation of China (32571643 and 82530116), the 1·3·5 Project for Disciplines of Excellence from West China Hospital, Sichuan University (ZYYC24009), and the Sichuan Province Science and Technology Major Special Project (2025ZDZX0039).

## Conflicts of Interest

The authors declare no conflicts of interest.

## Data Availability

The authors have nothing to report.

## References

[1] F. Bray , M. Laversanne , H. Sung , et al., “Global Cancer Statistics 2022: Globocan Estimates of Incidence and Mortality Worldwide for 36 Cancers in 185 Countries,” Cancer Journal for Clinicians 74, no. 3 (2024): 229–263. 10.3322/caac.21834 38572751

[2] M. Naghavi , H. H. Kyu , and A. Bhoomadevi , et al., “Global Burden of 292 Causes of Death in 204 Countries and Territories and 660 Subnational Locations, 1990–2023: A Systematic Analysis for the Global Burden of Disease Study 2023,” Lancet 406, no. 10513 (2025): 1811–1872. 41092928 10.1016/S0140-6736(25)01917-8 PMC12535838

[3] M. Gerlinger , A. J. Rowan , S. Horswell , et al., “Intratumor Heterogeneity and Branched Evolution Revealed by Multiregion Sequencing,” New England Journal of Medicine 366, no. 10 (2012): 883–892. 22397650 10.1056/NEJMoa1113205 PMC4878653

[4] J. Heyward , C. R. Lesko , J. C. Murray , et al., “Harm‐Benefit Balance of Immune Checkpoint Inhibitors in Non–Small Cell Lung Cancer,” JAMA Oncology 11, no. 7 (2025): 707–716. 40338588 10.1001/jamaoncol.2025.0985 PMC12062994

[5] M. J. Mitchell , M. M. Billingsley , R. M. Haley , et al., “Engineering Precision Nanoparticles for Drug Delivery,” Nature Reviews Drug Discovery 20, no. 2 (2021): 101–124. 33277608 10.1038/s41573-020-0090-8 PMC7717100

[6] P. Zhang , Y. Xiao , X. Sun , et al., “Cancer Nanomedicine toward Clinical Translation: Obstacles, Opportunities, and Future Prospects,” Medicine 4, no. 3 (2023): 147–167. 10.1016/j.medj.2022.12.001 36549297

[7] S. Hua , M. B. C. de Matos , J. M. Metselaar , et al., “Current Trends and Challenges in the Clinical Translation of Nanoparticulate Nanomedicines: Pathways for Translational Development and Commercialization,” Frontiers in Pharmacology 9 (2018): 1–14. 30065653 10.3389/fphar.2018.00790 PMC6056679

[8] B. Pelaz , C. Alexiou , R. A. Alvarez‐Puebla , et al., “Diverse Applications of Nanomedicine,” ACS Nano 11, no. 3 (2017): 2313–2381. 28290206 10.1021/acsnano.6b06040 PMC5371978

[9] G. Debnath , B. Vasu , and R. S. R. Gorla , “Current State‐of‐the‐Art in Multi‐Scale Modeling in Nano‐Cancer Drug Delivery: Role of AI and Machine Learning,” Cancer Nanotechnology 16, no. 1 (2025): 45.

[10] X. Shan , Y. Cai , B. Zhu , et al., “Rational Strategies for Improving the Efficiency of Design and Discovery of Nanomedicines,” Nature Communications 15, no. 1 (2024): 9990. 10.1038/s41467-024-54265-3 PMC11574076 39557860

[11] D. Q. Wang , L. Y. Feng , J. G. Ye , et al., “Accelerating the Integration of ChatGPT and Other Large‐Scale AI Models into Biomedical Research and Healthcare,” MedComm – Future Medicine 2, no. 2 (2023): 1–28.

[12] Y. Yu , G. Cai , R. Lin , et al., “Multimodal Data Fusion AI Model Uncovers Tumor Microenvironment Immunotyping Heterogeneity and Enhanced Risk Stratification of Breast Cancer,” MedComm 5, no. 12 (2024): e70023. 39669975 10.1002/mco2.70023 PMC11635117

[13] Y. Wu , N. Wang , P. Xiong , et al., “Artificial Intelligence for Drug Delivery: Yesterday, Today and Tomorrow,” Acta Pharmaceutica Sinica B (2025): 2068–2092. 42039289 10.1016/j.apsb.2025.09.022 PMC13104652

[14] N. I. Okafor , N. Igbokwe , H. Onohuean , et al., “Artificial Intelligence‐Driven Development and Characterization of Nanomedicine,” BioNanoScience 16, no. 4 (2026): 255.

[15] S. Azimi , “Intelligent Nanoparticle Design: Unlocking the Potential of AI for Transformative Drug Delivery,” Current Opinion in Biomedical Engineering 36 (2025): 100625.

[16] B. Patel , P. Darji , P. I. J. Fnu , et al., “A Comprehensive Review and Insight into the Latest Advancements in Nanotechnology,” Biosciences Biotechnology Research Asia 21, no. 3 (2024): 985–1000.

[17] A. Jahandoost , R. Dashti , M. Houshmand , et al., “Utilizing Machine Learning and Molecular Dynamics for Enhanced Drug Delivery in Nanoparticle Systems,” Scientific Reports 14, no. 1 (2024): 26677. 39496651 10.1038/s41598-024-73268-0 PMC11535187

[18] M. R. Kibria , R. I. Akbar , P. Nidadavolu , et al., “Predicting Efficacy of Drug‐Carrier Nanoparticle Designs for Cancer Treatment: A Machine Learning‐Based Solution,” Scientific Reports 13, no. 1 (2023): 547. 36631637 10.1038/s41598-023-27729-7 PMC9834306

[19] C. Shen , M. Zhang , M. Lu , et al., “Machine Learning Empowered Formulation Design, Optimization and Characterization of Nanoparticulate Drug Delivery Systems: Current Applications, Challenges, and Future Perspectives,” Acta Pharmaceutica Sinica B 16, no. 2 (2026): 665–685. 41685160 10.1016/j.apsb.2025.12.011 PMC12891881

[20] P. J. Dorsey , C. L. Lau , T. C. Chang , et al., “Review of Machine Learning for Lipid Nanoparticle Formulation and Process Development,” Journal of Pharmaceutical Sciences 113, no. 12 (2024): 3413–3433. 39341497 10.1016/j.xphs.2024.09.015

[21] L. Sun , H. Liu , Y. Ye , et al., “Smart Nanoparticles for Cancer Therapy,” Signal Transduction and Targeted Therapy 8, no. 1 (2023): 418. 37919282 10.1038/s41392-023-01642-x PMC10622502

[22] D. Killock , “Benefit of Nanomedicine Confirmed in Saml,” Nature Reviews Clinical Oncology 15, no. 10 (2018): 591. 10.1038/s41571-018-0080-5 30065313

[23] W. Chen , E. M. Goldys , and W. Deng , “Light‐Induced Liposomes for Cancer Therapeutics,” Progress in Lipid Research 79 (2020): 101052. 32679153 10.1016/j.plipres.2020.101052

[24] R. Rebollo , F. Oyoun , Y. Corvis , et al., “Microfluidic Manufacturing of Liposomes: Development and Optimization by Design of Experiment and Machine Learning,” ACS Applied Materials & Interfaces 14, no. 35 (2022): 39736–39745. 36001743 10.1021/acsami.2c06627

[25] R. Zwolsman , Y. B. Darwish , E. Kluza , et al., “Engineering Lipid Nanoparticles for mRNA Immunotherapy,” Nanomedicine and Nanobiotechnology 17, no. 2 (2025): e70007. 40195623 10.1002/wnan.70007 PMC11976204

[26] S. H. El Moukhtari , E. Garbayo , A. Amundarain , et al., “Lipid Nanoparticles for siRNA Delivery in Cancer Treatment,” Journal of Controlled Release 361 (2023): 130–146. 37532145 10.1016/j.jconrel.2023.07.054

[27] P. R. Cullis and P. L. Felgner , “The 60‐Year Evolution of Lipid Nanoparticles for Nucleic Acid Delivery,” Nature Reviews Drug Discovery 23 (2024): 709–722. 38965378 10.1038/s41573-024-00977-6

[28] S. Imani , X. Li , K. Chen , et al., “Computational Biology and Artificial Intelligence in mRNA Vaccine Design for Cancer Immunotherapy,” Frontiers in Cellular and Infection Microbiology 14 (2024): 1501010. 39902185 10.3389/fcimb.2024.1501010 PMC11788159

[29] Z. Liu , J. Chen , M. Xu , et al., “Engineered Multi‐Domain Lipid Nanoparticles for Targeted Delivery,” Chemical Society Reviews 54, no. 12 (2025): 5961–5994. 40390667 10.1039/d4cs00891j

[30] W. Wang , K. Chen , T. Jiang , et al., “Artificial Intelligence‐Driven Rational Design of Ionizable Lipids for mRNA Delivery,” Nature Communications 15, no. 1 (2024): 10804. 10.1038/s41467-024-55072-6 PMC11685617 39738043

[31] Y. Liao , X. Zeng , X. Zhang , et al., “Optimized Lipid Nanoparticles for Co‐Delivery of mRNA and siRNA Therapeutics in Refractory Liver Cancer,” Advanced Materials 38, no. 11 (2026): e19473. 41510588 10.1002/adma.202519473

[32] F. Danhier , E. Ansorena , J. M. Silva , et al., “PLGA‐Based Nanoparticles: An Overview of Biomedical Applications,” Journal of Controlled Release 161, no. 2 (2012): 505–522. 22353619 10.1016/j.jconrel.2012.01.043

[33] W. Gu , R. Qu , F. Meng , et al., “Polymeric Nanomedicines Targeting Hematological Malignancies,” Journal of Controlled Release 337 (2021): 571–588. 34364920 10.1016/j.jconrel.2021.08.001

[34] Y. Zhang , J. Chen , L. Shi , et al., “Polymeric Nanoparticle‐Based Nanovaccines for Cancer Immunotherapy,” Materials Horizons 10, no. 2 (2023): 361–392. 36541078 10.1039/d2mh01358d

[35] Z. Pei , H. Lei , and L. Cheng , “Bioactive Inorganic Nanomaterials for Cancer Theranostics,” Chemical Society Reviews 52, no. 6 (2023): 2031–2081. 36633202 10.1039/d2cs00352j

[36] B. B. Mendes , Z. Zhang , J. Conniot , et al., “A Large‐Scale Machine Learning Analysis of Inorganic Nanoparticles in Preclinical Cancer Research,” Nature Nanotechnology 19, no. 6 (2024): 867–878. 10.1038/s41565-024-01673-7 38750164

[37] F. Giulimondi , E. Vulpis , L. Digiacomo , et al., “Opsonin‐Deficient Nucleoproteic Corona Endows Unpegylated Liposomes With Stealth Properties in Vivo,” ACS Nano 16, no. 2 (2022): 2088–2100. 35040637 10.1021/acsnano.1c07687 PMC8867903

[38] S. Moayedi , W. Xia , L. Lundergan , et al., “Zwitterionic Polymers for Biomedical Applications: Antimicrobial and Antifouling Strategies toward Implantable Medical Devices and Drug Delivery,” Langmuir 40, no. 44 (2024): 23125–23145. 39450830 10.1021/acs.langmuir.4c02664

[39] B. Li , Z. Yuan , P. Jain , et al., “De Novo Design of Functional Zwitterionic Biomimetic Material for Immunomodulation,” Science Advances 6, no. 22 (2020): eaba0754. 32523997 10.1126/sciadv.aba0754 PMC7259941

[40] K. Sano , K. Umemoto , H. Miura , et al., “Feasibility of Using Poly[Oligo(Ethylene Glycol) Methyl Ether Methacrylate] as Tumor‐Targeted Carriers of Diagnostic Drugs,” ACS Applied Polymer Materials 4, no. 7 (2022): 4734–4740.

[41] A. Singh , N. Dowdall , and T. Hoare , “Poly(Oligo(Ethylene Glycol) Methacrylate)‐Based Polymers in Biomedical Applications: Preparation and Applications,” Biomacromolecules 26, no. 7 (2025): 3929–3973. 40464769 10.1021/acs.biomac.5c00145

[42] B. Sanchez‐Lengeling and A. Aspuru‐Guzik , “Inverse Molecular Design Using Machine Learning: Generative Models for Matter Engineering,” Science 361, no. 6400 (2018): 360–365. 30049875 10.1126/science.aat2663

[43] D. Menon and R. Ranganathan , “A Generative Approach to Materials Discovery, Design, and Optimization,” ACS Omega 7, no. 30 (2022): 25958–25973. 35936396 10.1021/acsomega.2c03264 PMC9352221

[44] D. M. Anstine and O. Isayev , “Generative Models as an Emerging Paradigm in the Chemical Sciences,” Journal of the American Chemical Society 145, no. 16 (2023): 8736–8750. 37052978 10.1021/jacs.2c13467 PMC10141264

[45] K. Chen , N. Fan , H. Huang , et al., “mRNA Vaccines Against SARS‐CoV‐2 Variants Delivered by Lipid Nanoparticles Based on Novel Ionizable Lipids,” Advanced Functional Materials 32, no. 39 (2022): 2204692. 35942272 10.1002/adfm.202204692 PMC9349794

[46] A. Tkatchenko , “Machine Learning for Chemical Discovery,” Nature Communications 11, no. 1 (2020): 4125. 10.1038/s41467-020-17844-8 PMC7431574 32807794

[47] S. Kim , C. M. Schroeder , and N. E. Jackson , “Open Macromolecular Genome: Generative Design of Synthetically Accessible Polymers,” ACS Polymers Au 3, no. 4 (2023): 318–330. 37576712 10.1021/acspolymersau.3c00003 PMC10416319

[48] S. Jiang and M. A. Webb , “Generative Active Learning Across Polymer Architectures and Solvophobicities for Targeted Rheological Behavior,” Computational Materials 12, no. 1 (2025): 28.

[49] L.‐J. Su , N.‐N. Wang , R. Luo , et al., “Artificial Intelligence‐Guided Design of LNPs for in Vivo Targeted mRNA Delivery via Analysis of the Spatial Conformation of Ionizable Lipids,” Nature Biomedical Engineering (2026): 1–22. 10.1038/s41551-026-01640-8 41851281

[50] K. Su , J. Qiu , T. Xu , et al., “Artificial Intelligence‐Guided Design of Lipid Nanoparticles for mRNA Delivery,” Acta Pharmaceutica Sinica B 16 (2026): 709–727. 41685167 10.1016/j.apsb.2025.11.029 PMC12891898

[51] B. Li , I. O. Raji , A. G. R. Gordon , et al., “Accelerating Ionizable Lipid Discovery for mRNA Delivery Using Machine Learning and Combinatorial Chemistry,” Nature Materials 23, no. 7 (2024): 1002–1008. 38740955 10.1038/s41563-024-01867-3

[52] J. Witten , I. Raji , R. S. Manan , et al., “Artificial Intelligence‐Guided Design of Lipid Nanoparticles for Pulmonary Gene Therapy,” Nature Biotechnology 43, no. 11 (2025): 1790–1799. 10.1038/s41587-024-02490-y PMC12149338 39658727

[53] R. Eugster , M. Orsi , G. Buttitta , et al., “Leveraging Machine Learning to Streamline the Development of Liposomal Drug Delivery Systems,” Journal of Controlled Release 376 (2024): 1025–1038. 39489466 10.1016/j.jconrel.2024.10.065

[54] A. Chan , A. R. Kirtane , Q. R. Qu , et al., “Designing Lipid Nanoparticles Using a Transformer‐Based Neural Network,” Nature Nanotechnology 20, no. 10 (2025): 1491–1501. 10.1038/s41565-025-01975-4 PMC12534189 40817189

[55] M. Sela , G. Chen , H. Kadosh , et al., “AI‐Validated Brain Targeted mRNA Lipid Nanoparticles With Neuronal Tropism,” ACS Nano 19, no. 41 (2025): 36106–36128. 40957853 10.1021/acsnano.4c15013 PMC12548354

[56] Y. Xu , S. Ma , H. Cui , et al., “Agile Platform: a Deep Learning Powered Approach to Accelerate LNP Development for mRNA Delivery,” Nature Communications 15, no. 1 (2024): 6305. 10.1038/s41467-024-50619-z PMC11282250 39060305

[57] N. Seegobin , Y. Abdalla , G. Li , et al., “Optimising the Production of PLGA Nanoparticles by Combining Design of Experiment and Machine Learning,” International Journal of Pharmaceutics 667 (2024): 124905. 39491656 10.1016/j.ijpharm.2024.124905

[58] A. Rahdar , S. Fathi‐Karkan , and M. Shirzad , “Integrating Machine Learning and Physics‐Based Modeling for Predictive Design of Gemcitabine‐Loaded Nanocomposites,” Scientific Reports (2026): 1–13. 10.1038/s41598-026-37098-6 PMC12905131 41593127

[59] J. Kehrein , A. Bunker , and R. Luxenhofer , “Poxload: Machine Learning Estimates Drug Loadings of Polymeric Micelles,” Molecular Pharmaceutics 21, no. 7 (2024): 3356–3374. 38805643 10.1021/acs.molpharmaceut.4c00086 PMC11394009

[60] P. Bannigan , Z. Bao , R. J. Hickman , et al., “Machine Learning Models to Accelerate the Design of Polymeric Long‐Acting Injectables,” Nature Communications 14, no. 1 (2023): 35. 10.1038/s41467-022-35343-w PMC9832011 36627280

[61] N. Shirokii , Y. Din , I. Petrov , et al., “Quantitative Prediction of Inorganic Nanomaterial Cellular Toxicity via Machine Learning,” Small 19, no. 19 (2023): e2207106. 36772908 10.1002/smll.202207106

[62] J. Cave , A. Christiono , C. Schiavone , et al., “Rational Design of Safer Inorganic Nanoparticles via Mechanistic Modeling‐Informed Machine Learning,” ACS Nano 19, no. 23 (2025): 21538–21555. 40460056 10.1021/acsnano.5c03590 PMC12177941

[63] S. Wilhelm , A. J. Tavares , Q. Dai , et al., “Analysis of Nanoparticle Delivery to Tumours,” Nature Reviews Materials 1, no. 5 (2016): 1–12.

[64] Q. Cheng , T. Wei , L. Farbiak , et al., “Selective Organ Targeting (Sort) Nanoparticles for Tissue‐Specific mRNA Delivery and CRISPR‐Cas Gene Editing,” Nature Nanotechnology 15, no. 4 (2020): 313–320. 10.1038/s41565-020-0669-6 PMC7735425 32251383

[65] N. Boehnke , J. P. Straehla , H. C. Safford , et al., “Massively Parallel Pooled Screening Reveals Genomic Determinants of Nanoparticle Delivery,” Science 377, no. 6604 (2022): eabm5551. 35862544 10.1126/science.abm5551 PMC10249039

[66] A. C. Marques , P. J. Costa , S. Velho , et al., “Functionalizing Nanoparticles With Cancer‐Targeting Antibodies: A Comparison of Strategies,” Journal of Controlled Release 320 (2020): 180–200. 31978444 10.1016/j.jconrel.2020.01.035

[67] T. L. Hunter , Y. Bao , Y. Zhang , et al., “In Vivo CAR T Cell Generation to Treat Cancer and Autoimmune Disease,” Science 388, no. 6753 (2025): 1311–1317. 40536974 10.1126/science.ads8473

[68] J. Rodriguez , A. De Iaco , J. B. Rottman , et al., “Targeted Lipid Nanoparticle Delivery of an RNA Gene Writer in Vivo Enables Generation of CAR‐T Cells in a Humanized Mouse Model,” Blood 144 (2024): 4799.

[69] A. Bot , A. Scharenberg , K. Friedman , et al., “In Vivo Chimeric Antigen Receptor (CAR)‐T Cell Therapy,” Nature Reviews Drug Discovery 25, no. 2 (2026): 116–137. 41028170 10.1038/s41573-025-01291-5

[70] L. DeFrancesco , “Genome Editing's Third Act,” Nature Biotechnology 44, no. 3 (2026): 331–333. 10.1038/s41587-026-03058-8 41844972

[71] H. Cai , Z. Zhang , M. Wang , et al., “Pretrainable Geometric Graph Neural Network for Antibody Affinity Maturation,” Nature Communications 15, no. 1 (2024): 7785. 10.1038/s41467-024-51563-8 PMC11379722 39242604

[72] N. R. Bennett , J. L. Watson , R. J. Ragotte , et al., “Atomically Accurate De Novo Design of Antibodies With RFdiffusion,” Nature 649, no. 8095 (2026): 183–193. 41193805 10.1038/s41586-025-09721-5 PMC12727541

[73] Q. Wang , X. Zhuang , J. Mu , et al., “Delivery of Therapeutic Agents by Nanoparticles Made of Grapefruit‐Derived Lipids,” Nature Communications 4 (2013): 1867. 10.1038/ncomms2886 PMC4396627 23695661

[74] Y. Li , T. N. Zamay , N. A. Luzan , et al., “Aptamers as a New Frontier in Targeted Cancer Therapy,” Advanced Drug Delivery Reviews 226 (2025): 115692. 41015208 10.1016/j.addr.2025.115692

[75] S. Venkatesan , K. Chanda , and M. M. Balamurali , “Recent Advancements of Aptamers in Cancer Therapy,” ACS Omega 8, no. 36 (2023): 32231–32243. 37720779 10.1021/acsomega.3c04345 PMC10500573

[76] A. Mullard , “FDA Approves Second RNA Aptamer,” Nature Reviews Drug Discovery 22, no. 10 (2023): 774. 10.1038/d41573-023-00148-z 37673979

[77] J. Hoellenriegel , D. Zboralski , C. Maasch , et al., “The Spiegelmer Nox‐A12, a Novel Cxcl12 Inhibitor, Interferes With Chronic Lymphocytic Leukemia Cell Motility and Causes Chemosensitization,” Blood 123, no. 7 (2014): 1032–1039. 24277076 10.1182/blood-2013-03-493924 PMC4123413

[78] P. J. Bates , D. A. Laber , D. M. Miller , et al., “Discovery and Development of the G‐Rich Oligonucleotide AS1411 as a Novel Treatment for Cancer,” Experimental and Molecular Pathology 86, no. 3 (2009): 151–164. 19454272 10.1016/j.yexmp.2009.01.004 PMC2716701

[79] E. M. Reyes‐Reyes , Y. Teng , and P. J. Bates , “A New Paradigm for Aptamer Therapeutic AS1411 Action: Uptake by Macropinocytosis and Its Stimulation by a Nucleolin‐Dependent Mechanism,” Cancer Research 70, no. 21 (2010): 8617–8629. 20861190 10.1158/0008-5472.CAN-10-0920 PMC2970734

[80] R. K. Kar , “High‐Throughput and Computational Techniques for Aptamer Design,” Expert Opinion on Drug Discovery 19, no. 12 (2024): 1457–1469. 39390781 10.1080/17460441.2024.2412632

[81] A. Bashir , Q. Yang , J. Wang , et al., “Machine Learning Guided Aptamer Refinement and Discovery,” Nature Communications 12, no. 1 (2021): 2366. 10.1038/s41467-021-22555-9 PMC8062585 33888692

[82] X. Yang , C. H. Chan , S. Yao , et al., “DeepAptamer: Advancing High‐Affinity Aptamer Discovery With a Hybrid Deep Learning Model,” Molecular Therapy—Nucleic Acids 36, no. 1 (2025): 102436. 39897584 10.1016/j.omtn.2024.102436 PMC11787022

[83] T. D. Thi Tran , H. L. Nguyen , P. T. Ngoc Hoa , et al., “Accelerated Aptamer Selection via SELEX and Molecular Simulations for Lipopolysaccharide Detection,” RSC Advances 15, no. 55 (2025): 46790–46806. 41323700 10.1039/d5ra06759f PMC12658737

[84] R. G. Jesky , L. H. Y. Lo , R. H. P. Siu , et al., “Designing the Future of Biosensing: Advances in Aptamer Discovery, Computational Modeling, and Diagnostic Applications,” Biosensors 15, no. 10 (2025): 1–54. 10.3390/bios15100637 PMC12562710 41149291

[85] A. L. Dias and T. Rodrigues , “Real‐World Validation of a Structure‐Aware Pipeline for Molecular Design,” Nature Machine Intelligence 7, no. 9 (2025): 1376–1377.

[86] M. Li , K. Song , J. He , et al., “Electron‐Density‐Informed Effective and Reliable De Novo Molecular Design and Optimization With ED2Mol,” Nature Machine Intelligence 7 (2025): 1355–1368.

[87] J. Guo , J. Chen , A. Gx‐Chen , et al., “Sample‐Efficient Generative Molecular Design Using Memory Manipulation,” Nature Machine Intelligence 8, no. 3 (2026): 449–460.

[88] L. Wang , Y. Wu , H. Luo , et al., “Learned Conformational Space and Pharmacophore into Molecular Foundational Model,” Advanced Science 13, no. 17 (2026): e13556. 41486619 10.1002/advs.202513556 PMC13042461

[89] X. Gou , S. He , B. Wang , et al., “Integrating PROTAC‐Based Targeted Protein Degradation With Nanodelivery Systems to Overcome Cancer Therapeutic Resistance,” Advanced Drug Delivery Reviews 229 (2026): 115755. 41386500 10.1016/j.addr.2025.115755

[90] Y. Liu , I. Ullah , Y. Yuan , et al., “Harnessing Targeted Protein Degradation to Potentiate Cancer Immunotherapy: from Molecular Mechanisms to Delivery Strategies,” Advanced Drug Delivery Reviews 230 (2026): 115776. 41525883 10.1016/j.addr.2026.115776

[91] P. Tan , S. Li , J. Huang , et al., “Harnessing Deep Learning to Accelerate the Development of Antibodies and Aptamers,” Acta Pharmaceutica Sinica B 16, no. 2 (2026): 788–801. 41685150 10.1016/j.apsb.2025.12.017 PMC12891845

[92] X. Peng , R. Guo , F. Guo , et al., “Unified Modeling of 3D Molecular Generation via Atomic Interactions With PocketXMol,” Cell 189, no. 7 (2026): 1904–1922. 41713417 10.1016/j.cell.2026.01.003

[93] F. Wu , Y. Zhao , J. Wu , et al., “De Novo Design of Epitope‐Specific Antibodies via a Structure‐Driven Computational Workflow,” Nature Communications 17, no. 1 (2025): 625. 10.1038/s41467-025-67361-9 PMC12815924 41365923

[94] J. A. Ruffolo , L.‐S. Chu , S. P. Mahajan , et al., “Fast, Accurate Antibody Structure Prediction from Deep Learning on Massive Set of Natural Antibodies,” Nature Communications 14, no. 1 (2023): 2389. 10.1038/s41467-023-38063-x PMC10129313 37185622

[95] D. Baizhigitova , I. E. Wu , L. Kalejaye , et al., “Molecular Modeling and Machine Learning for Predicting High‐Concentration Antibody Viscosity,” Advanced Drug Delivery Reviews 233 (2026): 115839. 41780732 10.1016/j.addr.2026.115839

[96] Y. Li , F. Wang , J. Yang , et al., “Deep Generative Optimization of mRNA Codon Sequences for Enhanced mRNA Translation and Therapeutic Efficacy,” Nature Communications 16, no. 1 (2025): 9957. 10.1038/s41467-025-64894-x PMC12612108 41224770

[97] H. Zhang , H. Liu , Y. Xu , et al., “Deep Generative Models Design mRNA Sequences With Enhanced Translational Capacity and Stability,” Science 390, no. 6773 (2025): eadr8470. 40875799 10.1126/science.adr8470

[98] A. K. Morrow , A. Thornal , E. D. Flynn , et al., “ML‐Driven Design of 3′ UTRs for mRNA Stability,” BioRxiv (2025): 1–27.

[99] L. Wang , S. Liu , J.‐X. Huang , et al., “Machine Learning‐Based Analysis of the Impact of 5′ Untranslated Region on Protein Expression,” Nucleic Acids Research 53, no. 17 (2025): gkaf861. 40923765 10.1093/nar/gkaf861 PMC12418383

[100] R. Long , Z. Guo , D. Han , et al., “siRNADiscovery: a Graph Neural Network for siRNA Efficacy Prediction via Deep RNA Sequence Analysis,” Briefings in Bioinformatics 25, no. 6 (2024): bbae563. 39503523 10.1093/bib/bbae563 PMC11539000

[101] Y. Bai , H. Zhong , T. Wang , et al., “Oligoformer: an Accurate and Robust Prediction Method for siRNA Design,” Bioinformatics 40, no. 10 (2024): btae577. 39321261 10.1093/bioinformatics/btae577 PMC11494384

[102] A. Boretti , “Synergizing Artificial Intelligence With in Vivo CAR‐T Cell Generation: A New Paradigm for Democratized Immunotherapy,” Bio Systems 262 (2026): 105750. 10.1016/j.biosystems.2026.105750 41763635

[103] S. M. Douglas , I. Bachelet , and G. M. Church , “A Logic‐Gated Nanorobot for Targeted Transport of Molecular Payloads,” Science 335, no. 6070 (2012): 831–834. 22344439 10.1126/science.1214081

[104] S. Li , Q. Jiang , S. Liu , et al., “A DNA Nanorobot Functions as a Cancer Therapeutic in Response to a Molecular Trigger in Vivo,” Nature Biotechnology 36, no. 3 (2018): 258–264. 10.1038/nbt.4071 29431737

[105] Y. Wang , I. Baars , I. Berzina , et al., “A DNA Robotic Switch With Regulated Autonomous Display of Cytotoxic Ligand Nanopatterns,” Nature Nanotechnology 19, no. 9 (2024): 1366–1374. 10.1038/s41565-024-01676-4 PMC11405282 38951595

[106] S. J. Park , S. H. Park , S. Cho , et al., “New Paradigm for Tumor Theranostic Methodology Using Bacteria‐Based Microrobot,” Scientific Reports 3 (2013): 3394. 24292152 10.1038/srep03394 PMC3844944

[107] A. Heidt , “How Nanobots Are Accelerating Cancer‐Targeting Therapies,” Nature 646, no. 8087 (2025): S39–S41. 41162724 10.1038/d41586-025-03480-z

[108] O. Felfoul , M. Mohammadi , S. Taherkhani , et al., “Magneto‐Aerotactic Bacteria Deliver Drug‐Containing Nanoliposomes to Tumour Hypoxic Regions,” Nature Nanotechnology 11, no. 11 (2016): 941–947. 10.1038/nnano.2016.137 PMC6094936 27525475

[109] L. Yang , J. Jiang , F. Ji , et al., “Machine Learning for Micro‐ and Nanorobots,” Nature Machine Intelligence 6, no. 6 (2024): 605–618.

[110] L. Yang , J. Jiang , X. Gao , et al., “Autonomous Environment‐Adaptive Microrobot Swarm Navigation Enabled by Deep Learning‐Based Real‐Time Distribution Planning,” Nature Machine Intelligence 4, no. 5 (2022): 480–493.

[111] C. K. Schmidt , M. Medina‐Sánchez , R. J. Edmondson , et al., “Engineering Microrobots for Targeted Cancer Therapies from a Medical Perspective,” Nature Communications 11, no. 1 (2020): 5618. 10.1038/s41467-020-19322-7 PMC7645678 33154372

[112] M. Medina‐Sánchez and O. G. Schmidt , “Medical Microbots Need Better Imaging and Control,” Nature 545, no. 7655 (2017): 406–408. 28541344 10.1038/545406a

[113] Z. Khan , N. Khan , M. Geetha , et al., “Therapeutic Applications of Nanobots and Nanocarriers in Cancer Treatment,” Analytical Sciences 41, no. 8 (2025): 1305–1324. 40465130 10.1007/s44211-025-00799-5 PMC12307532

[114] K. J. Hiam‐Galvez , B. M. Allen , and M. H. Spitzer , “Systemic Immunity in Cancer,” Nature Reviews Cancer 21, no. 6 (2021): 345–359. 33837297 10.1038/s41568-021-00347-z PMC8034277

[115] R. Strack , “Where the Nanocarriers Go,” Nature Biomedical Engineering 10, no. 2 (2026): 196. 10.1038/s41551-026-01623-9 41721104

[116] M. P. Monopoli , C. Aberg , A. Salvati , et al., “Biomolecular Coronas Provide the Biological Identity of Nanosized Materials,” Nature Nanotechnology 7 (2012): 779–786. 10.1038/nnano.2012.207 23212421

[117] M. Mahmoudi , M. P. Landry , A. Moore , et al., “The Protein Corona from Nanomedicine to Environmental Science,” Nature Reviews Materials (2023): 1–17. 37361608 10.1038/s41578-023-00552-2 PMC10037407

[118] E. Voke , M. L. Arral , H. J. Squire , et al., “Protein Corona Formed on Lipid Nanoparticles Compromises Delivery Efficiency of mRNA Cargo,” Nature Communications 16 (2025): 8699. 10.1038/s41467-025-63726-2 PMC12485112 41027853

[119] J. E. Blume , W. C. Manning , G. Troiano , et al., “Rapid, Deep and Precise Profiling of the Plasma Proteome With Multi‐Nanoparticle Protein Corona,” Nature Communications 11, no. 1 (2020): 3662. 10.1038/s41467-020-17033-7 PMC7376165 32699280

[120] R. N. Kularatne , R. M. Crist , and S. T. Stern , “The Future of Tissue‐Targeted Lipid Nanoparticle‐Mediated Nucleic Acid Delivery,” Pharmaceuticals 15, no. 7 (2022): 1–17. 10.3390/ph15070897 PMC9322927 35890195

[121] D. Paasch and N. Lachmann , “CAR Macrophages Tuning the Immune Symphony of Anti‐Cancer Therapies,” Cell Stem Cell 31, no. 6 (2024): 791–793. 38848684 10.1016/j.stem.2024.05.006

[122] J. Shen , S. Lyu , Y. Xu , et al., “Activating Innate Immune Responses Repolarizes hPSC‐Derived CAR Macrophages to Improve Anti‐Tumor Activity,” Cell Stem Cell 31, no. 7 (2024): 1003–1019. 38723634 10.1016/j.stem.2024.04.012

[123] Y. Wang , H. Zhao , and E. Cochran , “In Vivo Programming of Myeloid Cells by mRNA Mediated Delivery of Novel Fca Fusion Receptor Activates Anti‐Tumor Immunity,” (2022).

[124] X. Sun , M. D. Park , M. Merad , et al., “Macrophages: Targets for Next‐Generation Cancer Immunotherapy,” Cancer Cell 44, no. 3 (2026): 498–518. 41759521 10.1016/j.ccell.2026.01.020 PMC13331334

[125] J. Huzar , R. Coreas , M. P. Landry , et al., “AI‐Based Prediction of Protein Corona Composition on DNA Nanostructures,” ACS Nano 19, no. 4 (2025): 4333–4345. 39772513 10.1021/acsnano.4c12259 PMC11803750

[126] A. Canchola , K. Li , K. Chen , et al., “Meta‐Analysis and Machine Learning Prediction of Protein Corona Composition across Nanoparticle Systems in Biological Media,” ACS Nano 19, no. 43 (2025): 37633–37650. 41031442 10.1021/acsnano.5c08608 PMC12593346

[127] M. Mahmoudi , “The Need for Improved Methodology in Protein Corona Analysis,” Nature Communications 13, no. 1 (2022): 49. 10.1038/s41467-021-27643-4 PMC8748711 35013179

[128] Y. Zou , S. Ito , F. Yoshino , et al., “Polyglycerol Grafting Shields Nanoparticles from Protein Corona Formation to Avoid Macrophage Uptake,” ACS Nano 14, no. 6 (2020): 7216–7226. 32379425 10.1021/acsnano.0c02289

[129] K. Liu , R. Nilsson , E. Lázaro‐Ibáñez , et al., “Multiomics Analysis of Naturally Efficacious Lipid Nanoparticle Coronas Reveals High‐Density Lipoprotein Is Necessary for Their Function,” Nature Communications 14, no. 1 (2023): 4007. 10.1038/s41467-023-39768-9 PMC10325984 37414857

[130] S. A. Dilliard and D. J. Siegwart , “Passive, Active and Endogenous Organ‐Targeted Lipid and Polymer Nanoparticles for Delivery of Genetic Drugs,” Nature Reviews Materials 8, no. 4 (2023): 282–300. 36691401 10.1038/s41578-022-00529-7 PMC9850348

[131] W. C. Chou and Z. Lin , “Impact of Protein Coronas on Nanoparticle Interactions With Tissues and Targeted Delivery,” Current Opinion in Biotechnology 85 (2024): 103046. 38103519 10.1016/j.copbio.2023.103046 PMC11000521

[132] K. Gu , T. Liang , L. Hu , et al., “Intraperitoneal Programming of Tailored CAR Macrophages via mRNA Lipid Nanoparticle to Boost Cancer Immunotherapy,” Nature Communications 17, no. 1 (2025): 941. 10.1038/s41467-025-67674-9 PMC12830897 41444487

[133] X. Li , J. Zou , Z. He , et al., “The Interaction between Particles and Vascular Endothelium in Blood Flow,” Advanced Drug Delivery Reviews 207 (2024): 115216. 38387770 10.1016/j.addr.2024.115216

[134] M. Tollemeto and T. Lammers , “The Importance of Particle Shape in Drug Delivery,” Nature Reviews Bioengineering (2026): 388–390.

[135] A. Dasgupta , T. Sun , R. Palomba , et al., “Nonspherical Ultrasound Microbubbles,” Proceedings of the National Academy of Sciences 120, no. 13 (2023): e2218847120. 10.1073/pnas.2218847120 PMC10068850 36940339

[136] C. A. Mirkin , M. Mrksich , and N. Artzi , “The Emerging Era of Structural Nanomedicine,” Nature Reviews Bioengineering 3, no. 7 (2025): 526–528. 10.1038/s44222-025-00306-5 PMC12263095 40671716

[137] W. C. Chou , Q. Chen , L. Yuan , et al., “An Artificial Intelligence‐Assisted Physiologically‐Based Pharmacokinetic Model to Predict Nanoparticle Delivery to Tumors in Mice,” Journal of Controlled Release 361 (2023): 53–63. 37499908 10.1016/j.jconrel.2023.07.040 PMC11008607

[138] E. Gonzales‐Aloy , A. Ahmed‐Cox , M. Tsoli , et al., “From Cells to Organoids: The Evolution of Blood‐Brain Barrier Technology for Modelling Drug Delivery in Brain Cancer,” Advanced Drug Delivery Reviews 196 (2023): 114777. 36931346 10.1016/j.addr.2023.114777

[139] M. P. Ferro , S. C. Heilshorn , and R. M. Owens , “Materials for Blood‐Brain Barrier Modeling in Vitro,” Materials Science and Engineering: R: Reports 140 (2020): 100522. 33551572 10.1016/j.mser.2019.100522 PMC7864217

[140] A. Pérez‐López , A. I. Torres‐Suárez , C. Martín‐Sabroso , et al., “An Overview of in Vitro 3D Models of the Blood‐Brain Barrier as a Tool to Predict the in Vivo Permeability of Nanomedicines,” Advanced Drug Delivery Reviews 196 (2023): 114816. 37003488 10.1016/j.addr.2023.114816

[141] L. Dao , Z. You , L. Lu , et al., “Modeling Blood‐Brain Barrier Formation and Cerebral Cavernous Malformations in Human PSC‐Derived Organoids,” Cell Stem Cell 31, no. 6 (2024): 818–833. 38754427 10.1016/j.stem.2024.04.019 PMC11162335

[142] R. Ye , Y. Zhang , W. Xu , et al., “AI and Organoid Platforms for Brain‐Targeted Theranostics,” Theranostics 16, no. 2 (2026): 876–897. 41346712 10.7150/thno.123243 PMC12674940

[143] L. Bai and J. Su , “Artificial Intelligence Virtual Organoids (AIVOs),” Bioactive Materials 59 (2026): 45–68. 41536916 10.1016/j.bioactmat.2025.12.030 PMC12796111

[144] M. Binnewies , E. W. Roberts , K. Kersten , et al., “Understanding the Tumor Immune Microenvironment (Time) for Effective Therapy,” Nature Medicine 24, no. 5 (2018): 541–550. 10.1038/s41591-018-0014-x PMC5998822 29686425

[145] S. Sindhwani , A. M. Syed , J. Ngai , et al., “The Entry of Nanoparticles into Solid Tumours,” Nature Materials 19, no. 5 (2020): 566–575. 31932672 10.1038/s41563-019-0566-2

[146] H. Sabit , T. M. Pawlik , F. Radwan , et al., “Precision Nanomedicine: Navigating the Tumor Microenvironment for Enhanced Cancer Immunotherapy and Targeted Drug Delivery,” Molecular Cancer 24, no. 1 (2025): 160. 40457437 10.1186/s12943-025-02357-z PMC12131435

[147] J. Sheng , W. Yuan , M. Zhang , et al., “Overcoming Tumor Microenvironment Barriers: Transformable and Bioinspired Nanomedicine Strategies for Deep Tumor Penetration,” Journal of Nanobiotechnology (2026): 1–30. 41618406 10.1186/s12951-026-04028-7 PMC12931093

[148] M. Esposito , S. Ganesan , and Y. Kang , “Emerging Strategies for Treating Metastasis,” Nature Cancer 2, no. 3 (2021): 258–270. 33899000 10.1038/s43018-021-00181-0 PMC8064405

[149] S. Ye , S. Chen , V. Basava , et al., “And Logic Nanoparticle for Precision Immunotherapy of Metastatic Cancers,” Nature Nanotechnology 21, no. 4 (2026): 606–616. 10.1038/s41565-026-02130-3 PMC13106039 41803437

[150] M. Malhotra and A. Kulkarni , “Logic‑Gated Nanomedicine Activates STING to Boost Metastatic Tumour Immunotherapy,” Nature Nanotechnology (2026): 488–489. 10.1038/s41565-026-02131-2 41803436

[151] C. A. Klebanoff , H. T. Khong , P. A. Antony , et al., “Sinks, Suppressors and Antigen Presenters: How Lymphodepletion Enhances T Cell‐Mediated Tumor Immunotherapy,” Trends in Immunology 26, no. 2 (2005): 111–117. 15668127 10.1016/j.it.2004.12.003 PMC1388277

[152] C. M. Schurch , S. S. Bhate , G. L. Barlow , et al., “Coordinated Cellular Neighborhoods Orchestrate Antitumoral Immunity at the Colorectal Cancer Invasive Front,” Cell 182, no. 5 (2020): 1341–1359. 32763154 10.1016/j.cell.2020.07.005 PMC7479520

[153] W. A. Mahdi , A. Alhowyan , and A. J. Obaidullah , “Intelligence Analysis of Drug Nanoparticles Delivery Efficiency to Cancer Tumor Sites Using Machine Learning Models,” Scientific Reports 15, no. 1 (2025): 1017. 39762427 10.1038/s41598-024-84450-9 PMC11704265

[154] P. MacMillan , A. M. Syed , B. R. Kingston , et al., “Toward Predicting Nanoparticle Distribution in Heterogeneous Tumor Tissues,” Nano Letters 23, no. 15 (2023): 7197–7205. 37506224 10.1021/acs.nanolett.3c02186

[155] K. Mi , W. C. Chou , Q. Chen , et al., “Predicting Tissue Distribution and Tumor Delivery of Nanoparticles in Mice Using Machine Learning Models,” Journal of Controlled Release 374 (2024): 219–229. 39146980 10.1016/j.jconrel.2024.08.015 PMC11886896

[156] J. Luo , M. Molbay , Y. Chen , et al., “Nanocarrier Imaging at Single‐Cell Resolution across Entire Mouse Bodies With Deep Learning,” Nature Biotechnology 43 (2025): 2009–2022. 10.1038/s41587-024-02528-1 PMC12700832 39809933

[157] Z. Lin , W. C. Chou , Y. H. Cheng , et al., “Predicting Nanoparticle Delivery to Tumors Using Machine Learning and Artificial Intelligence Approaches,” International Journal of Nanomedicine 17 (2022): 1365–1379. 35360005 10.2147/IJN.S344208 PMC8961007

[158] W. C. Chou , A. Canchola , F. Zhang , et al., “Machine Learning and Artificial Intelligence in Nanomedicine,” Nanomedicine and Nanobiotechnology 17, no. 4 (2025): e70027. 40813104 10.1002/wnan.70027 PMC12353477

[159] S. N. Bhatia , X. Chen , M. A. Dobrovolskaia , et al., “Cancer Nanomedicine,” Nature Reviews Cancer 22, no. 10 (2022): 550–556. 10.1038/s41568-022-00496-9 PMC9358926 35941223

[160] J. Shi , P. W. Kantoff , R. Wooster , et al., “Cancer Nanomedicine: Progress, Challenges and Opportunities,” Nature Reviews Cancer 17, no. 1 (2017): 20–37. 27834398 10.1038/nrc.2016.108 PMC5575742

[161] J. Nam , S. Son , K. S. Park , et al., “Cancer Nanomedicine for Combination Cancer Immunotherapy,” Nature Reviews Materials 4, no. 6 (2019): 398–414.

[162] R. Baskaran , J. Lee , and S. G. Yang , “Clinical Development of Photodynamic Agents and Therapeutic Applications,” Biomaterials Research 22 (2018): 25. 30275968 10.1186/s40824-018-0140-z PMC6158913

[163] D. Fan , Y. Cao , M. Cao , et al., “Nanomedicine in Cancer Therapy,” Signal Transduction and Targeted Therapy 8, no. 1 (2023): 293. 37544972 10.1038/s41392-023-01536-y PMC10404590

[164] V. Mittelheisser , V. Gensbittel , L. Bonati , et al., “Evidence and Therapeutic Implications of Biomechanically Regulated Immunosurveillance in Cancer and Other Diseases,” Nature Nanotechnology 19, no. 3 (2024): 281–297. 10.1038/s41565-023-01535-8 38286876

[165] B. Wang , S. Hu , Y. Teng , et al., “Current Advance of Nanotechnology in Diagnosis and Treatment for Malignant Tumors,” Signal Transduction and Targeted Therapy 9, no. 1 (2024): 200. 39128942 10.1038/s41392-024-01889-y PMC11323968

[166] W. Jiang , Y. Wang , J. A. Wargo , et al., “Considerations for Designing Preclinical Cancer Immune Nanomedicine Studies,” Nature Nanotechnology 16, no. 1 (2021): 6–15. 10.1038/s41565-020-00817-9 PMC8103921 33349682

[167] N. Andor , T. A. Graham , M. Jansen , et al., “Pan‐Cancer Analysis of the Extent and Consequences of Intratumor Heterogeneity,” Nature Medicine 22, no. 1 (2016): 105–113. 10.1038/nm.3984 PMC4830693 26618723

[168] R. K. Jain and T. Stylianopoulos , “Delivering Nanomedicine to Solid Tumors,” Nature Reviews Clinical Oncology 7, no. 11 (2010): 653–664. 10.1038/nrclinonc.2010.139 PMC3065247 20838415

[169] A. T. Florence , “Targeting″ Nanoparticles: the Constraints of Physical Laws and Physical Barriers,” Journal of Controlled Release 164 (2012): 115–124. 22484196 10.1016/j.jconrel.2012.03.022

[170] M. Y. Want , A. Konstorum , R. Y. Huang , et al., “Neoantigens Retention in Patient Derived Xenograft Models Mediates Autologous T Cells Activation in Ovarian Cancer,” Oncoimmunology 8, no. 6 (2019): e1586042. 31069153 10.1080/2162402X.2019.1586042 PMC6492964

[171] T. S. Hauck , R. E. Anderson , H. C. Fischer , et al., “In Vivo Quantum‐Dot Toxicity Assessment,” Small 6 (2010): 138–144. 19743433 10.1002/smll.200900626

[172] R. L. Siegel , K. D. Miller , and A. Jemal , “Cancer Statistics, 2020,” Cancer Journal for Clinicians 70 (2020): 7–30. 10.3322/caac.21590 31912902

[173] H. Forgham , L. Liu , T. P. Davis , et al., “Antifouling Surface Coatings for the Next Generation of Nanomedicine: Toward in Vivo Immune Evasion,” Nanomedicine 18, no. 27 (2023): 1997–2000. 37982577 10.2217/nnm-2023-0316

[174] J. Yang , X. Wang , B. Wang , et al., “Challenging the Fundamental Conjectures in Nanoparticle Drug Delivery for Chemotherapy Treatment of Solid Cancers,” Advanced Drug Delivery Reviews 190 (2022): 114525. 36100142 10.1016/j.addr.2022.114525

[175] M. A. Younis , H. M. Tawfeek , A. A. H. Abdellatif , et al., “Clinical Translation of Nanomedicines: Challenges, Opportunities, and Keys,” Advanced Drug Delivery Reviews 181 (2022): 114083. 34929251 10.1016/j.addr.2021.114083

[176] X. Ma , Y. Tian , R. Yang , et al., “Nanotechnology in Healthcare, and Its Safety and Environmental Risks,” Journal of Nanobiotechnology 22, no. 1 (2024): 715. 39548502 10.1186/s12951-024-02901-x PMC11566612

[177] C. Domingues , A. Santos , C. Alvarez‐Lorenzo , et al., “Where Is Nano Today and Where Is It Headed? A Review of Nanomedicine and the Dilemma of Nanotoxicology,” ACS Nano 16, no. 7 (2022): 9994–10041. 35729778 10.1021/acsnano.2c00128

[178] X. Ma , M. Mao , J. He , et al., “Nanoprobe‐Based Molecular Imaging for Tumor Stratification,” Chemical Society Reviews 52, no. 18 (2023): 6447–6496. 37615588 10.1039/d3cs00063j

[179] D. Strepkos , M. Markouli , A. Klonou , et al., “Insights in the Immunobiology of Glioblastoma,” Journal of Molecular Medicine 98, no. 1 (2020): 1–10. 31650201 10.1007/s00109-019-01835-4

[180] S. A. Moosavian , V. Bianconi , M. Pirro , et al., “Challenges and Pitfalls in the Development of Liposomal Delivery Systems for Cancer Therapy,” Seminars in Cancer Biology 69 (2021): 337–348. 31585213 10.1016/j.semcancer.2019.09.025

[181] R. Kantesaria and H. S. Panda , “A Review on AI‐Based Data‐Driven Models for Optimization of Nanocarriers as Drug Delivery Systems,” ACS Biomaterials Science & Engineering 12, no. 3 (2026): 1397–1418. 41668335 10.1021/acsbiomaterials.5c01998

[182] J. Suksaeree , “A Review of Artificial Intelligence (AI)‐Driven Smart and Sustainable Drug Delivery Systems: A Dual‐Framework Roadmap for the Next Pharmaceutical Paradigm,” Sci 7, no. 4 (2025): 179.

[183] Y. A. Fahim , I. W. Hasani , S. Kabba , et al., “Artificial Intelligence in Healthcare and Medicine: Clinical Applications, Therapeutic Advances, and Future Perspectives,” European Journal of Medical Research 30, no. 1 (2025): 848. 40988064 10.1186/s40001-025-03196-w PMC12455834

[184] C. Guan , B. B. Mendes , J. Conniot , et al., “Accelerating Discoveries in Cancer Nanomedicine Using AI,” Cell Biomaterials 1, no. 11 (2025): 100279.

[185] O. M. Sarhan , M. I. Gebril , and D. Elsegaie , “Artificial Intelligence‐Enabled Nanomedicine: Enhancing Drug Design and Predictive Modeling in Pharmaceutics,” Journal of Pharmacy and Pharmacology 78, no. 3 (2026): rgaf113. 41353571 10.1093/jpp/rgaf113

[186] M. Faria , M. Björnmalm , K. J. Thurecht , et al., “Minimum Information Reporting in Bio–Nano Experimental Literature,” Nature Nanotechnology 13, no. 9 (2018): 777–785. 10.1038/s41565-018-0246-4 PMC6150419 30190620

[187] J. G. Greener , S. M. Kandathil , L. Moffat , et al., “A Guide to Machine Learning for Biologists,” Nature Reviews Molecular Cell Biology 23, no. 1 (2022): 40–55. 34518686 10.1038/s41580-021-00407-0

[188] D. M. Camacho , K. M. Collins , R. K. Powers , et al., “Next‐Generation Machine Learning for Biological Networks,” Cell 173, no. 7 (2018): 1581–1592. 29887378 10.1016/j.cell.2018.05.015

[189] M. D. Wilkinson , M. Dumontier , I. J. Aalbersberg , et al., “The Fair Guiding Principles for Scientific Data Management and Stewardship,” Scientific Data 3 (2016): 160018. 26978244 10.1038/sdata.2016.18 PMC4792175

[190] L. Elberskirch , K. Binder , N. Riefler , et al., “Digital Research Data: from Analysis of Existing Standards to a Scientific Foundation for a Modular Metadata Schema in Nanosafety,” Particle and Fibre Toxicology 19, no. 1 (2022): 1. 34983569 10.1186/s12989-021-00442-x PMC8728981

[191] N. Jeliazkova , E. Longhin , N. El Yamani , et al., “A Template Wizard for the Cocreation of Machine‐Readable Data‐Reporting to Harmonize the Evaluation of (Nano)Materials,” Nature Protocols 19, no. 9 (2024): 2642–2684. 38755447 10.1038/s41596-024-00993-1

[192] J. Hastings , N. Jeliazkova , G. Owen , et al., “Enanomapper: Harnessing Ontologies to Enable Data Integration for Nanomaterial Risk Assessment,” Journal of Biomedical Semantics 6 (2015): 10. 25815161 10.1186/s13326-015-0005-5 PMC4374589

[193] H. Zhao , Y. Wang , A. Lin , et al., “Nanomine Schema: an Extensible Data Representation for Polymer Nanocomposites,” APL Materials 6, no. 11 (2018): 111108.

[194] M. Faria , M. Bjornmalm , K. J. Thurecht , et al., “Minimum Information Reporting in Bio–Nano Experimental Literature,” Nature Nanotechnology 13, no. 9 (2018): 777–785. 10.1038/s41565-018-0246-4 PMC6150419 30190620

[195] M. Abolhasani and E. Kumacheva , “The Rise of Self‐Driving Labs in Chemical and Materials Sciences,” Nature Synthesis 2, no. 6 (2023): 483–492.

[196] E. Stach , B. DeCost , A. G. Kusne , et al., “Autonomous Experimentation Systems for Materials Development: A Community Perspective,” Matter 4, no. 9 (2021): 2702–2726.

[197] J. Peng , D. Schwalbe‐Koda , K. Akkiraju , et al., “Human‐ and Machine‐Centred Designs of Molecules and Materials for Sustainability and Decarbonization,” Nature Reviews Materials 7, no. 12 (2022): 991–1009.

[198] B. P. MacLeod , F. G. L. Parlane , T. D. Morrissey , et al., “Self‐Driving Laboratory for Accelerated Discovery of Thin‐Film Materials,” Science Advances 6, no. 20 (2020): eaaz8867. 32426501 10.1126/sciadv.aaz8867 PMC7220369

[199] R. J. Hickman , P. Bannigan , Z. Bao , et al., “Self‐Driving Laboratories: A Paradigm Shift in Nanomedicine Development,” Matter 6, no. 4 (2023): 1071–1081. 37020832 10.1016/j.matt.2023.02.007 PMC9993483

[200] B. A. Koscher , R. B. Canty , M. A. McDonald , et al., “Autonomous, Multiproperty‐Driven Molecular Discovery: From Predictions to Measurements and Back,” Science 382, no. 6677 (2023): eadi1407. 38127734 10.1126/science.adi1407

[201] Y. Xu , H. Cui , K. Pang , et al., “Lumi‐Lab: A Foundation Model‐Driven Autonomous Platform Enabling Discovery of Ionizable Lipid Designs for mRNA Delivery,” Cell 189, no. 6 (2026): 1620–1635. 41742414 10.1016/j.cell.2026.01.012

[202] S. Sharifi , N. Reuel , N. Kallmyer , et al., “The Issue of Reliability and Repeatability of Analytical Measurement in Industrial and Academic Nanomedicine,” ACS Nano 17, no. 1 (2023): 4–11. 36573831 10.1021/acsnano.2c09249 PMC10546893

[203] M. F. Dockendorf , B. J. Hansen , K. P. Bateman , et al., “Digitally Enabled, Patient‐Centric Clinical Trials: Shifting the Drug Development Paradigm,” Clinical and Translational Science 14, no. 2 (2021): 445–459. 33048475 10.1111/cts.12910 PMC7993267

[204] E. J. Austin , C. LeRouge , J. R. Lee , et al., “A Learning Health Systems Approach to Integrating Electronic Patient‐Reported Outcomes across the Health Care Organization,” Learning Health Systems 5, no. 4 (2021): e10263. 34667879 10.1002/lrh2.10263 PMC8512814

[205] M. Li , L. Li , P. Yang , et al., “A Machine Learning‐Enhanced Gastric Cancer Diagnostic Method Based on Shell‐Isolated Nanoparticle‐Enhanced Raman Spectroscopy,” Nanoscale 17, no. 45 (2025): 26214–26224. 41081475 10.1039/d5nr02377g

[206] J. Ko , N. Bhagwat , S. S. Yee , et al., “Combining Machine Learning and Nanofluidic Technology to Diagnose Pancreatic Cancer Using Exosomes,” ACS Nano 11, no. 11 (2017): 11182–11193. 29019651 10.1021/acsnano.7b05503

[207] H. Sharma , V. Yadav , and C. D'Souza‐Schorey , “A Scalable High‐Throughput Isoelectric Fractionation Platform for Extracellular Nanocarriers: Comprehensive and Bias‐Free Isolation of Ribonucleoproteins from Plasma, Urine, and Saliva,” ACS Nano 17, no. 10 (2023): 9388–9404. 37071723 10.1021/acsnano.3c01340 PMC10756736

[208] Y. Wen , Y. Li , S. Cheng , et al., “Partition‐Less Digital Immunoassay Using Configurable Topographic Nanoarrays for Extracellular Vesicle Diagnosis of Ewing Sarcoma,” ACS Nano 19, no. 12 (2025): 11973–11986.40115997 10.1021/acsnano.4c16904

[209] T. Hu , H. Xiao , S. Ji , et al., “An Artificial Intelligence‐Enhanced Early Ovarian Cancer Diagnosis Biosensor,” Journal of Materials Chemistry B 13, no. 37 (2025): 11696–11707. 40878196 10.1039/d5tb00993f

[210] A. Ing , A. Andrades , M. R. Cosenza , et al., “Integrating Multimodal Cancer Data Using Deep Latent Variable Path Modelling,” Nature Machine Intelligence 7, no. 7 (2025): 1053–1075. 10.1038/s42256-025-01052-4 PMC12283373 40709098

[211] F. Chen , H. Chen , T. Yu , et al., “AI‐Driven Revolution of Medical Robotics across Surgical Innovation, Rehabilitation Intelligence, and Multimodal Healthcare Delivery,” MedComm 7, no. 3 (2026): e70597. 41810390 10.1002/mco2.70597 PMC12968330

[212] J. Lv , L. X. Xu , Z. X. Li , et al., “Longitudinal on‐Treatment Circulating Tumor DNA as a Biomarker for Real‐Time Dynamic Risk Monitoring in Cancer Patients: the Ep‐Season Study,” Cancer Cell 42, no. 8 (2024): 1401–1414. 39059389 10.1016/j.ccell.2024.07.001

[213] Z. Obermeyer , “Bedside to Bench—AI and the New Science of Medicine,” The New England Journal of Medicine 393, no. 23 (2025): 2287–2289. 41358600 10.1056/NEJMp2510203

[214] V. Chugh , A. Basu , A. Kaushik , et al., “Employing Nano‐Enabled Artificial Intelligence (AI)‐Based Smart Technologies for Prediction, Screening, and Detection of Cancer,” Nanoscale 16, no. 11 (2024): 5458–5486. 38391246 10.1039/d3nr05648a

[215] S. J. Greive , L. Bacri , B. Cressiot , et al., “Identification of Conformational Variants for Bradykinin Biomarker Peptides from a Biofluid Using a Nanopore and Machine Learning,” ACS Nano 18, no. 1 (2024): 539–550. 38134312 10.1021/acsnano.3c08433

[216] K. S. Kapoor , S. Kong , H. Sugimoto , et al., “Single Extracellular Vesicle Imaging and Computational Analysis Identifies Inherent Architectural Heterogeneity,” ACS Nano 18, no. 18 (2024): 11717–11731. 38651873 10.1021/acsnano.3c12556 PMC12002403

[217] S. Pal , D. Huttner , N. C. Verma , et al., “Amplification‐Free Quantification of Endogenous Mitochondrial DNA Copy Number Using Solid‐State Nanopores,” ACS Nano 19, no. 11 (2025): 11390–11402. 40082088 10.1021/acsnano.5c00732 PMC11948453

[218] U. Parlatan , M. O. Ozen , I. Kecoglu , et al., “Label‐Free Identification of Exosomes Using Raman Spectroscopy and Machine Learning,” Small 19, no. 9 (2023): e2205519. 36642804 10.1002/smll.202205519

[219] L. Min , H. Bao , F. Bu , et al., “Machine‐Learning‐Assisted Procoagulant Extracellular Vesicle Barcode Assay toward High‐Performance Evaluation of Thrombosis‐Induced Death Risk in Cancer Patients,” ACS Nano 17, no. 20 (2023): 19914–19924. 37791763 10.1021/acsnano.3c04615

[220] J. Chi , Y. Xue , Y. Zhou , et al., “Perovskite Probe‐Based Machine Learning Imaging Model for Rapid Pathologic Diagnosis of Cancers,” ACS Nano 18, no. 35 (2024): 24295–24305. 39164203 10.1021/acsnano.4c06351

[221] S. Lee , M. Jue , K. Lee , et al., “Early‐Stage Diagnosis of Bladder Cancer Using Surface‐Enhanced Raman Spectroscopy Combined With Machine Learning Algorithms in a Rat Model,” Biosensors and Bioelectronics 246 (2024): 115915. 38081101 10.1016/j.bios.2023.115915

[222] D. Ishwar , R. Haldavnekar , S. Das , et al., “Glioblastoma Associated Natural Killer Cell EVs Generating Tumour‐Specific Signatures: Noninvasive GBM Liquid Biopsy With Self‐Functionalized Quantum Probes,” ACS Nano 16, no. 7 (2022): 10859–10877. 35816089 10.1021/acsnano.2c03055

[223] S. Premachandran , R. Haldavnekar , S. Das , et al., “Deep Surveillance of Brain Cancer Using Self‐Functionalized 3D Nanoprobes for Noninvasive Liquid Biopsy,” ACS Nano 16, no. 11 (2022): 17948–17964. 36112671 10.1021/acsnano.2c04187

[224] S. Premachandran , I. Shreshtha , K. Venkatakrishnan , et al., “Detection of Brain Metastases from Blood Using Brain Nanomet Sensor: Extracellular Vesicles as a Dynamic Marker for Metastatic Brain Tumors,” Biosensors and Bioelectronics 269 (2025): 116968. 39586755 10.1016/j.bios.2024.116968

[225] N. Cheng , Y. Gao , S. Ju , et al., “Serum Analysis Based on SERS Combined With 2D Convolutional Neural Network and Gramian Angular Field for Breast Cancer Screening,” Spectrochimica Acta Part A, Molecular and Biomolecular Spectroscopy 312 (2024): 124054. 38382221 10.1016/j.saa.2024.124054

[226] X. Feng , W. Zhang , and S. Chang‐Hao Tsao , “Integrated Chemical Array and SERS Profiling of Plasma Small Extracellular Vesicles for Breast Cancer Diagnosis,” Nano Letters 25, no. 42 (2025): 15331–15339. 41084822 10.1021/acs.nanolett.5c04034

[227] D. Ishwar , S. Premachandran , S. Das , et al., “Profiling Breast Tumor Heterogeneity and Identifying Breast Cancer Subtypes through Tumor‐Associated Immune Cell Signatures and Immuno Nano Sensors,” Small 20, no. 52 (2024): e2406475. 39460487 10.1002/smll.202406475 PMC11673452

[228] T. M. Nguyen , S. Jeong , S. K. Kang , et al., “3D Superclusters With Hybrid Bioinks for Early Detection in Breast Cancer,” ACS Sensors 9, no. 2 (2024): 699–707. 38294962 10.1021/acssensors.3c01938 PMC10897927

[229] T. M. Nguyen , T. M. T. Nguyen , S.‐J. Kim , et al., “Microcapillary‐Derived Plasmonic‐Enhanced Cluster through the Self‐Assembly Process for Breast Cancer Diagnosis,” ACS Sensors 10, no. 4 (2025): 2589–2597. 39929749 10.1021/acssensors.4c03051

[230] D. Ishwar , K. Venkatakrishnan , B. Tan , et al., “DNA Methylation Signatures of Tumor‐Associated Natural Killer Cells With Self‐Functionalized Nanosensor Enable Colorectal Cancer Diagnosis,” Nano Letters 23, no. 10 (2023): 4142–4151. 37134017 10.1021/acs.nanolett.2c04914

[231] C. Li , Z. Wang , P. Ding , et al., “A Digital Score Based on Circulating‐Tumor‐Cells‐Derived mRNA Quantification and Machine Learning for Early Colorectal Cancer Detection,” ACS Nano 19, no. 19 (2025): 18117–18128. 40335073 10.1021/acsnano.4c15056

[232] H. Chen , C. Huang , Y. Wu , et al., “Exosome Metabolic Patterns on Aptamer‐Coupled Polymorphic Carbon for Precise Detection of Early Gastric Cancer,” ACS Nano 16, no. 8 (2022): 12952–12963. 35946596 10.1021/acsnano.2c05355

[233] Z. Liu , T. Li , Z. Wang , et al., “Gold Nanopyramid Arrays for Non‐Invasive Surface‐Enhanced Raman Spectroscopy‐Based Gastric Cancer Detection via sEVs,” ACS Applied Nano Materials 5, no. 9 (2022): 12506–12517. 36185166 10.1021/acsanm.2c01986 PMC9513748

[234] X. Li , Y. Liu , Y. Fan , et al., “Advanced Nanoencapsulation‐Enabled Ultrasensitive Analysis: Unraveling Tumor Extracellular Vesicle Subpopulations for Differential Diagnosis of Hepatocellular Carcinoma via DNA Cascade Reactions,” ACS Nano 18, no. 17 (2024): 11389–11403. 38628141 10.1021/acsnano.4c01310

[235] D. Chen , Y. Wang , Y. Wei , et al., “Size‐Coded Hydrogel Microbeads for Extraction‐Free Serum Multi‐miRNAs Quantifications With Machine‐Learning‐Aided Lung Cancer Subtypes Classification,” Nano Letters 25, no. 1 (2025): 453–460. 39680719 10.1021/acs.nanolett.4c05233

[236] S. Ganesh , P. Dharmalingam , S. Das , et al., “Mapping Immune‐Tumor Bidirectional Dialogue Using Ultrasensitive Nanosensors for Accurate Diagnosis of Lung Cancer,” ACS Nano 17, no. 9 (2023): 8026–8040. 37093561 10.1021/acsnano.2c09323

[237] V. T. N. Linh , H. Kim , M.‐Y. Lee , et al., “3D Plasmonic Hexaplex Paper Sensor for Label‐Free Human Saliva Sensing and Machine Learning‐Assisted Early‐Stage Lung Cancer Screening,” Biosensors and Bioelectronics 244 (2024): 115779. 37922808 10.1016/j.bios.2023.115779

[238] Z. Liu , J. Zhang , N. Wang , et al., “Enhanced YOLOv5 Network‐Based Object Detection (Balfilter Reader) Promotes Perfect Filter‐Enabled Liquid Biopsy of Lung Cancer from Bronchoalveolar Lavage Fluid (BALF),” Microsystems & Nanoengineering 9 (2023): 121. 37786899 10.1038/s41378-023-00580-6 PMC10541878

[239] V. T. N. Linh , M.‐Y. Lee , J. Mun , et al., “3D Plasmonic Coral Nanoarchitecture Paper for Label‐Free Human Urine Sensing and Deep Learning‐Assisted Cancer Screening,” Biosensors and Bioelectronics 224 (2023): 115076. 36641876 10.1016/j.bios.2023.115076

[240] J. Chen , X. Yu , Y. Qu , et al., “High‐Performance Metabolic Profiling of High‐Risk Thyroid Nodules by ZrMOF Hybrids,” ACS Nano 18, no. 32 (2024): 21336–21346. 39090798 10.1021/acsnano.4c05700

[241] S. Park , Y. Jo , S.‐J. Kim , et al., “Fabrication of Serum‐Based SERS‐Tailored 3D Structures for Thyroid Cancer Diagnosis,” Scientific Reports 15, no. 1 (2025): 22262. 40594741 10.1038/s41598-025-06346-6 PMC12216531

[242] J. K. Hemmingsen , M. H. Enemark , E. F. Sørensen , et al., “Proteomic Profiling Identifies Predictive Signatures for Progression Risk in Patients With Advanced‐Stage Follicular Lymphoma,” Cancers 16, no. 19 (2024): 3278. 39409899 10.3390/cancers16193278 PMC11476298

[243] U.S. Food and Drug Administration Considerations for the Use of Artificial Intelligence to Support Regulatory Decision‐Making for Drug and Biological Products: Draft Guidance for Industry and Other Interested Parties (U.S. Food and Drug Administration, 2025).

[244] S. M. Lundberg and S.‐I. Lee , “A Unified Approach to Interpreting Model Predictions,” in Proceedings of the 31st International Conference on Neural Information Processing Systems (Curran Associates Inc., 2017).

[245] J. Pearl , Causality: Models, Reasoning, and Inference, 2 ed (Cambridge University Press, 2009), 10.1017/CBO9780511803161.

[246] Medicines and Healthcare Products Regulatory Agency , Impact of AI on the Regulation of Medical Products: Implementing the AI White Paper Principles. (2024): 1–17.

[247] L. K. Vora , A. D. Gholap , K. Jetha , et al., “Artificial Intelligence in Pharmaceutical Technology and Drug Delivery Design,” Pharmaceutics 15, no. 7 (2023): 1–46. 10.3390/pharmaceutics15071916 PMC10385763 37514102

[248] C. M. Dawidczyk , L. M. Russell , and P. C. Searson , “Recommendations for Benchmarking Preclinical Studies of Nanomedicines,” Cancer Research 75, no. 19 (2015): 4016–4020. 26249177 10.1158/0008-5472.CAN-15-1558 PMC4592474

[249] K. T. Butler , D. W. Davies , H. Cartwright , et al., “Machine Learning for Molecular and Materials Science,” Nature 559, no. 7715 (2018): 547–555. 30046072 10.1038/s41586-018-0337-2

[250] V. Tshitoyan , J. Dagdelen , L. Weston , et al., “Unsupervised Word Embeddings Capture Latent Knowledge from Materials Science Literature,” Nature 571, no. 7763 (2019): 95–98. 31270483 10.1038/s41586-019-1335-8

[251] M. Alber , A. Buganza Tepole , W. R. Cannon , et al., “Integrating Machine Learning and Multiscale Modeling‐Perspectives, Challenges, and Opportunities in the Biological, Biomedical, and Behavioral Sciences,” NPJ Digital Medicine 2 (2019): 115. 31799423 10.1038/s41746-019-0193-y PMC6877584

[252] A. Letai , “Functional Precision Cancer Medicine‐Moving beyond Pure Genomics,” Nature Medicine 23, no. 9 (2017): 1028–1035. 10.1038/nm.4389 28886003

[253] M. A. Miller , S. Gadde , C. Pfirschke , et al., “Predicting Therapeutic Nanomedicine Efficacy Using a Companion Magnetic Resonance Imaging Nanoparticle,” Science Translational Medicine 7, no. 314 (2015): 314ra183. 10.1126/scitranslmed.aac6522 PMC5462466 26582898

[254] S. M. McKinney , M. Sieniek , V. Godbole , et al., “International Evaluation of an AI System for Breast Cancer Screening,” Nature 577, no. 7788 (2020): 89–94. 31894144 10.1038/s41586-019-1799-6

[255] P. Tan , X. Chen , H. Zhang , et al., “Artificial Intelligence Aids in Development of Nanomedicines for Cancer Management,” Seminars in Cancer Biology 89 (2023): 61–75. 36682438 10.1016/j.semcancer.2023.01.005

[256] C. J. Kelly , A. Karthikesalingam , M. Suleyman , et al., “Key Challenges for Delivering Clinical Impact With Artificial Intelligence,” BMC Medicine 17, no. 1 (2019): 195. 31665002 10.1186/s12916-019-1426-2 PMC6821018

[257] J. R. Zech , M. A. Badgeley , M. Liu , et al., “Variable Generalization Performance of a Deep Learning Model to Detect Pneumonia in Chest Radiographs: a Cross‐Sectional Study,” PLOS Medicine 15, no. 11 (2018): e1002683. 30399157 10.1371/journal.pmed.1002683 PMC6219764

[258] H. Zhang , R. Song , F. Guo , et al., “Using Physical Organic Chemistry Knowledge to Predict Unusual Metabolites of Synthetic Phenolic Antioxidants by Cytochrome P450,” Chemical Research in Toxicology 35, no. 5 (2022): 840–848. 35416036 10.1021/acs.chemrestox.2c00021

[259] A. Trezza , A. Visibelli , B. Roncaglia , et al., “Unsupervised Learning in Precision Medicine: Unlocking Personalized Healthcare through AI,” Applied Sciences 14, no. 20 (2024): 1–17.

[260] R. Bonazzola , E. Ferrante , N. Ravikumar , et al., “Unsupervised Ensemble‐Based Phenotyping Enhances Discoverability of Genes Related to Left‐Ventricular Morphology,” Nature Machine Intelligence 6, no. 3 (2024): 291–306. 10.1038/s42256-024-00801-1 PMC10957472 38523678

[261] S. Azizi , L. Culp , J. Freyberg , et al., “Robust and Data‐Efficient Generalization of Self‐Supervised Machine Learning for Diagnostic Imaging,” Nature Biomedical Engineering 7, no. 6 (2023): 756–779. 10.1038/s41551-023-01049-7 37291435

[262] S. Kulkarni , B. Lin , and R. Radhakrishnan , “Machine Learning Enabled Multiscale Model for Nanoparticle Margination and Physiology Based Pharmacokinetics,” Computers & Chemical Engineering 198 (2025): 109081. 40657536 10.1016/j.compchemeng.2025.109081 PMC12245191

[263] L. Mollica , C. Leli , F. Sottotetti , et al., “Digital Twins: a New Paradigm in Oncology in the Era of Big Data,” ESMO Real World Data and Digital Oncology 5 (2024): 100056. 41648658 10.1016/j.esmorw.2024.100056 PMC12836754

[264] M. De Domenico , L. Allegri , G. Caldarelli , et al., “Challenges and Opportunities for Digital Twins in Precision Medicine from a Complex Systems Perspective,” Digital Medicine 8, no. 1 (2025): 37. 39825012 10.1038/s41746-024-01402-3 PMC11742446

[265] I. Karaman and B. Sebin , “From Data‐Driven Cities to Data‐Driven Tumors: Dynamic Digital Twins for Adaptive Oncology,” Frontiers in Artificial Intelligence 8 (2025): 1624877. 40785836 10.3389/frai.2025.1624877 PMC12331622

[266] D. Singh , S. Singh , and N. Tandon , “From Bench to Bedside: Role of Digital Twins in Nanocarrier Pharmacokinetics and Biodistribution,” Nano LIFE (2025): 1–20.

[267] R. van der Meel , E. Sulheim , Y. Shi , et al., “Smart Cancer Nanomedicine,” Nature Nanotechnology 14, no. 11 (2019): 1007–1017. 10.1038/s41565-019-0567-y PMC7227032 31695150

[268] J.‐N. May , J. I. Moss , F. Mueller , et al., “Histopathological Biomarkers for Predicting the Tumour Accumulation of Nanomedicines,” Nature Biomedical Engineering 8, no. 11 (2024): 1366–1378. 10.1038/s41551-024-01197-4 PMC7616664 38589466

[269] F. Man , T. Lammers , and R. T. M. de Rosales , “Imaging Nanomedicine‐Based Drug Delivery: a Review of Clinical Studies,” Molecular Imaging and Biology 20, no. 5 (2018): 683–695. 30084044 10.1007/s11307-018-1255-2 PMC6139024

[270] Q. Yin , A. Pan , B. Chen , et al., “Quantitative Imaging of Intracellular Nanoparticle Exposure Enables Prediction of Nanotherapeutic Efficacy,” Nature Communications 12, no. 1 (2021): 2385. 10.1038/s41467-021-22678-z PMC8062465 33888701

[271] H. Abdollahi , F. Yousefirizi , I. Shiri , et al., “Theranostic Digital Twins: Concept, Framework and Roadmap towards Personalized Radiopharmaceutical Therapies,” Theranostics 14, no. 9 (2024): 3404–3422. 38948052 10.7150/thno.93973 PMC11209714

